# Plasma and Serum LC-MS Lipidomic Fingerprints of Bipolar Disorder and Schizophrenia

**DOI:** 10.3390/ijms26136134

**Published:** 2025-06-26

**Authors:** Marija Takić, Vesna Jovanović, Suzana Marković, Zoran Miladinović, Milka Jadranin, Gordana Krstić, Čedo Miljević, Vele Tešević, Boris Mandić

**Affiliations:** 1 Group for Nutrition and Metabolism, Center of Research Excellence for Nutrition and Metabolism, Institute for Medical Research, National Institute of Republic of Serbia, University of Belgrade, Dr Subotića 4, 11000 Belgrade, Serbia; 2 University of Belgrade-Faculty of Chemistry, Studentski trg 12–16, 11000 Belgrade, Serbia; vjovanovic@chem.bg.ac.rs (V.J.); suzana.markovic@med.bg.ac.rs (S.M.); gkrstic@chem.bg.ac.rs (G.K.); vtesevic@chem.bg.ac.rs (V.T.); 3 University of Belgrade-Faculty of Medicine, Institute of Forensic Medicine, Deligradska 31a, 11000 Belgrade, Serbia; 4 Institute of General and Physical Chemistry, Studentski trg 12–16, 11158 Belgrade, Serbia; 5 Department of Chemistry, University of Belgrade-Institute of Chemistry, Technology and Metallurgy, Njegoševa 12, 11000 Belgrade, Serbia; milka.jadranin@ihtm.bg.ac.rs; 6 University of Belgrade-Faculty of Medicine, dr Subotića 8, 11000 Belgrade, Serbia; cedo.miljevic@yahoo.com

**Keywords:** biomarker, lipids, phospholipids, sphingolipids, ceramides

## Abstract

Bipolar disorder (BD) and schizophrenia (SCH) are results of the complex interactions between genetic and environmental factors, and the underlying pathophysiology is not yet completely understood. The current diagnostic criteria for psychiatric diagnosis are based purely on clinical phenomenology and they are limited to psychiatrist judgment after a standardized clinical interview, with no precise biomarkers used to discriminate between the disorders. Besides gaps in the understanding and diagnosis of these diseases, there is also a need for personalized and precise approaches to patients through customized medical treatment and reliable monitoring of treatment response. To fulfill existing gaps, the establishment of disorder biomarker sets is a necessary step. LC-MS lipidomic blood sample analysis is one of the ongoing omics approaches. In the last ten years, several studies have identified alterations in lipid metabolism associated with BD and SCH, and this review summarizes current knowledge on their lipidomic patterns, which is essential for identifying lipid biomarkers. Currently, findings indicate decreases in plasmalogens and acyl-carnitines, along with increases in certain triacylglycerol species, shared by both conditions. In contrast, serum LC-MS lipidomic profiles of sphingolipids including ceramides could be unique to BD, indicating the need for further investigation in future studies.

## 1. Introduction

Schizophrenia (SCH) and mood disorders are not entirely separate conditions and can occur simultaneously. Psychotic episodes may arise within mood disorders, while mood symptoms can also be present in primary psychotic disorders [1]. Some researchers suggest that these conditions exist along a continuum rather than being completely distinct, with varying symptom severity and characteristics between the two extremes [2]. The similarity in epidemiological data (i.e., age at onset, lifetime risk, worldwide distribution, risk for suicide) supports the hypothesis of a continuum between schizophrenia and mood disorders [3]. This dimensional perspective contrasts with the categorical approach, which considers these disorders as separate entities with distinct causes and underlying mechanisms [4]. Disorders on the psychosis–mood spectrum include schizophrenia, bipolar disorder (BD), paranoia, and schizoaffective disorder. The biomarkers from the same group of psychiatric conditions are more likely to be similar and may overlap. Therefore, exploring and comparing across disorders on the psychosis–mood spectrum is crucial to ensure the diagnosis specifics of potential biomarkers or a set of biomarkers. However, to the best of our knowledge, there are no reported lipidomics studies exploring blood lipid biomarkers of schizoaffective disorder and/or paranoia; for that reason, this review explores only LC-MS lipidomics studies of SCH and BD patients.

SCH and BD are widespread mental disorders ranked in the top 25 causes of disability worldwide, and lead to significant disabilities, functional impairments and premature death. The international prevalence estimation of SCH among non-institutionalized persons is 0.33% to 0.75% [5]; in the European Union population, the estimated incidence of schizophrenia is 15.2 per 100,000 persons [6], and in the U.S., the prevalence of SCH ranges between 0.25% and 0.64% [7]. The lifetime prevalence of bipolar disorders is estimated at 3.9% in a US population and data from the UK, Germany, and Italy indicated a prevalence of bipolar disorder of ~1% [8]. Global total costs of these disorders are enormous. The total costs of mental health problems are estimated at more than 4% of GDP (more than EUR 600 billion) across the 27 EU countries. US total yearly costs are estimated at over USD 300 billion for SCH and BD. Economic burden of psychosis mood spectrum disorders varies from 0.02% to 1.65% of the gross domestic product through low-, medium-, and high-income countries, with indirect cost contributions of 50–85% [9,10,11].

BD is a complex and severe mental health condition marked by episodes of mood and energy fluctuations, ranging from manic to depressive episodes. This chronic illness can also manifest psychotic symptoms, such as delusions and hallucinations. In most cases, BD patients initially experience a depressive episode, where symptoms differ slightly from unipolar depression [12]. BD often emerges in adolescence or early adulthood, causing lifelong functional impairments, encompassing cognitive difficulties, alongside a heightened risk of premature death [12,13,14]. At the same time, current drug treatments involving antipsychotics and antidepressants are not effective in all BD patients and can induce serious side effects [15,16].

SCH is a chronic psychiatric illness that affects cognition, perception, emotions, and behavior, leading to disability, invalidity, and is associated with a 15–20-year shorter life expectancy for patients with SCH compared to the general population [17,18]. The initial symptoms typically arise during adolescence or early adulthood [19], making early diagnosis and appropriate treatment crucial. SCH shares several symptoms like hallucinations, delusions, disorganized thinking and behavior, extreme agitation, emotional flatness, cognitive symptoms, social withdraw, and negative syndromes including a lack of motivation, reduced emotional expression, and a lack of anhedonia with other psychiatric disorders: BD, depression, anxiety, obsessive–compulsive disorders, and autism [14,20].

To date, there is no available instrumental clinical test based on blood analyses for any mental disorder. The diagnosis of BD and SCH is primarily based on a constellation of symptoms, their duration, and the exclusion of other potential causes. The evaluation of patients is conducted through clinical assessments according to specific diagnostic criteria during interviews between the doctor and the patients and/or their caretakers. The discovery of biomarkers could enable the development of objective clinical tests, leading to earlier, more accurate, and precise diagnoses, as well as the monitoring of mental disorders, ultimately slowing their progression. Due to overlapping symptoms in BD and SCH, distinguishing between these two disorders and establishing an accurate diagnosis is a huge challenge, but this is crucial for effective treatment. A lipid biomarker determination could be incorporated as a complementary test, a screening tool, and to predict and follow up on treatment response.

Lipids serve different physiological functions, including the assembly of cellular membranes, the storage of metabolic energy, and the signaling molecules in different cell processes such as proliferation, differentiation, apoptosis, and inflammation [21,22,23,24]. As downstream biochemical end-products, lipid metabolites are more closely related to phenotypes than to genomics and proteomics [25]. At the same time, lipids and molecules associated with lipid metabolism are known to play direct and causal roles in the pathophysiology of various human diseases, including cardiovascular, metabolic, chronic inflammations, and neuropsychiatric diseases [24,26,27,28]. Since the alterations in lipids proceed with the onset of neuropsychiatric diseases, these molecules may be promising biomarkers for the diagnosis, treatment, and prognosis of BD and SCH. The possibility of using lipids as markers has emerged because of the great abundance of lipids in the human brain [29], the close relationship between lipid metabolism and neuropsychiatric diseases [28], and the lipid-altering effects of medications used to treat such disorders [30]. Furthermore, recent genome-wide association studies (GWASs) have uncovered an overlap between susceptibility loci for neuropsychiatric disorders such as SCH and BD, and genes linked to the regulation of lipid levels, not exploring effects of genes on lipid profiles, but demonstrating statistical correlations [31]. Numerous studies indicate that the dysregulation of the metabolism of glycerophospholipids (GPs), lipid peroxidation due to oxidative stress, and the synthesis of lipid pro/anti-inflammatory mediators from polyunsaturated fatty acids (PUFAs) are of great importance for the development and progression of these diseases [28,32,33,34,35]. The crucial factor for the brain tissues that are susceptible to oxidative stress is its high oxygen consumption as well as enrichment with unsaturated FAs [36,37]. The influence of external variables such as diet, obesity, medication use, and other comorbidities associated with psychiatric disorders is a critical factor for biomarker validity, too. Although current scientific research is focused on understanding how these factors impact lipidomics profiles [38,39,40], our knowledge remains limited at this moment. To enhance the identification of validated biomarkers, further lipidomics studies in psychiatric patients have to be carefully designed to account for limitations arising from these factors.

**Figure 1:**
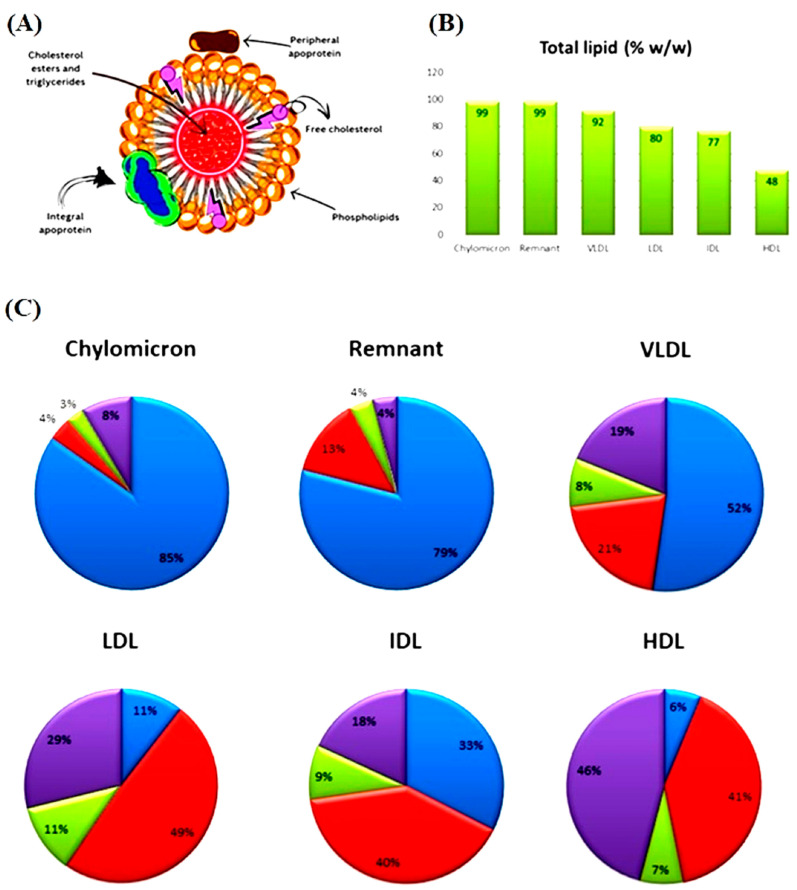
Structure and lipid distribution of serum/plasma lipoprotein particles: (**A**) schematic presentation of a structure of a lipoprotein particle; (**B**) total lipid amounts of major types of serum/plasma lipoprotein particles; (**C**) lipid composition of major serum/plasma types of lipoprotein particles (blue—triacyclglycerols, red—cholesterol esters, green—cholesterol, purple—phospholipids). Source for Figure 1B,C: adapted from Hone et al. [52].

Lipidomics and metabolomics have recently been developed as promising approaches to understand lipid biology that could facilitate the discovery of lipid biomarkers for various psychiatric disorders, including BD and SCH [41,42]. The main advantages of lipidomic analysis, typically utilizing HPLC-MS-based technology, are that it requires small amounts of biological samples, allows the measurement of thousands of different molecules in a single run, and has the highest sensitivity compared to other omics methods [43,44]. However, several important limitations remain to be addressed before it can be used as a routine tool in clinical practice and research. These include ensuring the reproducibility of measurements, the standardization of an analytical procedure, implementing internal and external quality control to assessed test sensitivity and specificity, and establishing reference values and intervals considering age and sex as key determinants [38,45,46]. In addition, genetic and various environmental factors including diet, obesity, gut microbiome, ethnicity, use of drugs/medications, and lifestyle contribute to interindividual variability [38]. These factors pose limitations beside analytical barriers that contribute to a lack of reproducibility between lipidomics studies. Finally, the high cost of instrumentation and sample analysis presents a significant challenge that limits the widespread use of lipidomics (https://www.lipotype.com, accessed on 15 May 2025). The LIPID MAPS Consortium reported the quantitative levels of over 500 lipid species in human reference plasma, revealing a remarkable diversity of plasma lipids from the six major lipid classes: free fatty acids (FFAs), glycerolipids (GLs), GPs, sphingolipids (SLs), sterols (STs), and prenols (PRs) [47]. Of GLs, the authors quantified levels of 18 triacylglycerols (TG) species and of 55 diacylglycerol (DGs) species, including both 1,2- and 1,3-DGs, and the total concentration of TGs was approximately 20 times higher than that of DGs. In this study [47], the following GPs were identified: phosphatidylethanolamine (PE), lysophosphatidylethanolamine (LPE), phosphatidylcholine (PC), lysophosphatidylcholine (LPC), phosphatidylserine (PS), phosphatidylglycerol (PG), phosphatidic acid (PA), phosphatidylinositol (PI), and N-acyl-PS. The SL class included sphingomyelins (SMs), monohexosylceramides, ceramides (Cers), and sphingoid bases, whereas dolichols and coenzyme Q were measured as representatives from a group of prenol lipids. Additionally, in some of lipidomic studies included in this review, the applied LC-MS technology enabled differentiation between diacyl- and ether (alkylacyl-) GPs (abbreviation PX(O-)) [32,41]. Specific sub-groups of ether GPs (alkylacyl-phospholipids), usually called plasmalogens, with a vinyl group on the sn-1 position were also identified in some of the lipidomic studies (abbreviation PX(P-)) [48,49], as well as acyl-carnitines (acyl-CARs) [49,50]. Guo et al. (2022) [51] have identified 31 lipid classes in plasma, including (O-acyl)-1-hydroxy FAs, wax esters (WEs), digalactosyldiacylglycerols (DGDGs), monogalactosyldiacylglycerols (MGDGs), monoacylglycerols (MAGs), dihexosyl N acetylhexosyl ceramide (CerG2GNAc1), ganglioside GM3, phytosphingomyelin (phSM), Ceramide-1-phosphate (CerP), ganglioside GD2, ganglioside GM2, lysophosphatidylinositol (LPI), phosphatidylinositol phosphates (PIP), and phosphatidylinositol biphosphates (PIP2), beside those lipid classes that were identified in the LIPID MAPS Consortium study and other mentioned studies [47,48,49,50]. In circulation, lipids are packed in lipoprotein particles including chylomicrons, remnants, very-low-density lipoproteins (VLDLs), intermediate-density lipoproteins (IDLs), low-density lipoproteins (LDLs), and high-density lipoproteins (HDLs), and their structure and lipid compositions are presented in Figure 1.

There have been several earlier reviews focusing on lipid markers in SCH, particularly regarding n-3 polyunsaturated fatty acids (PUFAs) [53,54] and products of lipid peroxidation [55,56]. In several review articles, the authors discussed studies on the endocannabinoid system in SCH, too [57]. Recently, a systematic review on lipidomic biomarkers of brain and peripheral materials in patients with SCH was published [58], and Wu et al. (2024) provided an overview of data regarding peripheral lipid signatures in SCH spectrum disorders [59]. Regarding patients with BD, Hiller et al. (2023) published a review article on the topic of lipid markers, focusing on serum lipoproteins and FAs [60]. To the best of our knowledge, no reviews have addressed serum and plasma lipidomic biomarkers of mood spectrum psychosis, including SCH and BD, of which symptoms are commonly overlapping. Thus, the main objective of this article is to provide a comprehensive review of this topic and address possible similarities and differences in lipidomic fingerprints of BD and SCH.

## 2. Materials and Methods

The PubMed and Scopus search was conducted from 15 November 2024 until 12 February 2025 (Figure 1), using keywords “lipidomics and schizophrenia”, “lipidomics and bipolar disorder”, “lipidomics and schizoaffective disorders”, and “schizophrenia and paranoia”, with the goal of preparing an overview of the current knowledge regarding serum and plasma lipidomics profiles in mood spectrum psychosis lipidomic studies. Based on the search results, as there were no findings for lipidomics related to schizoaffective disorders and paranoia, the review on SCH and BD was prepared. A search for the keywords “lipidomics and bipolar disorder” returned 26 publications in the PubMed database and 17 publications in the Scopus database over the last ten years, of which 12 were common results in both databases. In terms of the search for “lipidomics and schizophrenia”, a greater number of publications were identified, with 56 found in the PubMed search and 43 found in the Scopus search, of which 28 were shared between the two databases. Only human cohort studies utilizing the LC-MS method to measure plasma and serum lipids, and studies in which the obtained lipid profiles in neuropsychiatric diseases were compared with those of healthy control groups were selected for this review. The review has focused on LC-MS-based studies, as this methodology is most frequently used and considered valid for this purpose. However, the exclusion of other lipidomic techniques (e.g., NMR) remains a methodological limitation of the review’s scope. In the first step, we excluded book chapters, editorials, review articles, and articles not written in English. The remaining original articles written in English included studies involving animal models, those focused on drug treatment without reporting lipidomics data for the control group, and studies analyzing other types of materials (such as brain tissue, erythrocytes, platelets, urine, and plasma exosomes). The two studies focusing on the association of weight gain with lipidomics profiles were also excluded, as they did not include control subjects. The number of lipidomics studies is rapidly increasing and only a few serum and plasma lipidomics studies on SCH were published prior to 2014. Overall, based on PubMed and Scopus searches, 15 articles fulfilled all criteria and were included in this review: 5 focused on BD patients (Section 3.1), 8 focused on SCH patients (Section 3.2), and 2 included both BD and SCH patients (Section 3.1, Section 3.2 and Section 3.3).

## 3. Serum and Plasma Lipidomics Fingerprints of Bipolar Disorder and Schizophrenia

### 3.1. Serum and Plasma Lipidomics Studies in Patients with BD

Numerous studies provide evidence of lipid metabolism alterations including the metabolism of arachidonic acid, GPs, GLs, and SLs related to BD [42,61,62]. In a recent review by Hiller et al. (2023) [60], the authors observed that in 21 out of 38 studies that analyzed total-, HDL cholesterol, LDL cholesterol, or triglycerides, at least one of these parameters was changed in BD patients compared to controls. The authors also reported that the most consistent findings considering FA profiles of erythrocytes, serum, and plasma were decreases in the contents of EPA and DHA in the patients. Finally, among the nine omics studies discussed in the review, four reported changes in GPs, three found alterations in GL contents, and three observed differences in FFA composition. In the last decade, the use of both targeted and untargeted lipidomics approaches to identify alterations in lipid profiles, and thus potential biomarkers of psychiatric diseases, has been increasing. A systematic search of the available literature of studies published over the past decade enabled the identification of seven studies which examined serum and plasma lipidomic profiles in BD patients versus healthy controls using LC-MS-based platforms (Table 1). The comparison of the results of these studies represents a challenge due to the heterogeneity of measured lipids considering the number of lipids species and lipid classes identified. At this moment, there is no accredited method for the analysis of lipidomics profiles of serum and plasma, and different methodological approaches are employed for the preparation of samples and LC-MS analysis by each research group. Together with the use of different laboratory equipment, this resulted in significant variability in the set of identified serum and plasma lipid metabolites. The aforementioned factors could be among the main reasons why further lipidomics studies in patients with BD are needed. Future studies should use a standardized methodology to analyze profiles in larger numbers of recruited drug-naïve patients as well as patients on drug treatments, while also considering data about their diet, genetics, weight, lipid status, insulin sensitivity, and similar variables, to overcome the limitations of present studies.

Ribeiro et al. (2017) [63] performed ultra-high-performance liquid chromatography (UHPLC)-MS analysis to identify potential biomarkers that could distinguish BD type I patients and healthy controls with a negative history of psychiatric disorders. The blood samples were collected in the morning after overnight fasting, and serum lipid profiles were analyzed in 14 patients with BD and 21 age-, sex-, and BMI-matched healthy controls aged 18 to 65 years. Recruited BD patients were euthymic and their Yang Mania Rating Scale (YMRS) and 17-items Hamilton Depression Scale (HDRS-17) scores were expectantly low. Most of the identified lipid species belonged to the following lipid classes: GPs, SPs, FFAs, and GLs. In the BD group, the percentage of GPs decreased, while that of SLs increased compared to the control group. The 121 differential lipids were identified by comparing the BD group to the healthy control group, reflecting alterations in lipid metabolism, particularly in the GP class. Ribeiro et al. (2017) measured that 63 serum lipid species were upregulated, and 58 were downregulated in the BD group compared to the healthy control group [63]. The lipid metabolites that contributed the most to the separation of groups were putatively identified molecules: PI (40:3), PG (32:4(OH)), PA (48:8(OH)), PA (44:4), PE (42:5), and TG (42:3).

The study conducted by Brunkhorst-Kanaan et al. (2019) [64] used both targeted and untargeted lipidomic approaches to identify alterations in plasma lipid metabolites in unipolar and BD patients compared to the healthy control group. Their main research focus was on molecules belonging to lipid class SLs (including Cer) and lysophosphatidic acids, as well as endocannabinoids and pterins. The plasma lipid profiles of 67 patients of both genders, older than 18 years, with unipolar or bipolar disorders, and 405 age-, gender-, and BMI-matched healthy controls were determined. Compared to the control group, the amounts of ceramides (Cer16:0, Cer18:0, Cer20:0, Cer22:0, Cer24:1) and hexosyl- and dihexosyl-metabolites of Cer24:1 (GluCer24:1, LacCer24:1) were increased in the depression group (both unipolar and bipolar patients). Among other factors, Cer levels were associated with age, plasma TG and DG levels, and the use of antidepressants and olanzapine (antipsychotic). In contrast, there was no statistically significant association between the severity of symptoms and plasma Cer levels.

In a plasma untargeted lipidomic study [51], the blood samples of 54 non-obese females (24 with BD aged from 22 to 58 years, and 30 age- and BMI-matched healthy controls) were collected in the morning under fasting conditions and analyzed by an LC-MS/MS platform. Comprehensive lipid profiling was provided and a quantitative report on the composition of various lipids was given. A total of 31 lipid classes and 884 lipid species were identified in both groups. Compared with the control group, in BD patients, concentrations of several lipid classes were upregulated and downregulated. The concentrations of WEs, acyl-CARs, SMs, coenzyme Q, MGDG, PS, and PE were significantly lower in the BD group compared to healthy controls. By contrast, their PI, LPC, LPE, LPI, PIP, PIP2, Cer, CerP, GD2, GM2, TG, and MG concentrations were significantly higher. These changes in lipid profiles were associated with the symptoms and severity of BD evaluated by the administered the Hamilton Depression Rating Scale (HAMD), the Hamilton Anxiety Scale (HAMA), the Positive and Negative Syndrome Scale (PANSS), and Bech-Rafaelson Mania Rating Scale (BRMS) tests. The ROC analysis has revealed that changes in acyl-CARs and LPE might be the promising candidates for BD diagnosis biomarkers. The carbon chain lengths and degree of unsaturation of the FAs in the identified lipids also differed between the patient group and the control group. The levels of long-chain FAs with 16, 17, and more than 44 carbon atoms were increased, whereas the levels of those with 20–24, 33, 35, 36, 41, and 43 were decreased in the BD group. Moreover, saturated FAs (SFAs), monounsaturated FAs (MUFAs), and PUFAs with five, eight, and nine double bonds were increased in BD patients. In contrast, the levels of PUFAs with six and more than nine double bonds were decreased in the patient group, compared to healthy controls. Finally, the concentrations of 55 lipid species were significantly altered in the BD group compared to the control group, with 12 species showing a decrease and 43 species showing an increase. The panel of nine differential lipids including PS (42:9), DG (21:5), PC (36:6), PC (8:0/6:0), PS (16:1/22:4), TG (16:0/16:1/22:6), TG (16:0/20:4/22:5), TG (22:4/17:1/18:2), and TG (18:0/8:0/20:4) was found to give an optimal classification performance with an area under curve (AUC) value of 0.994 in ROC analysis.

Using the same LC-MS/MS-based methodological approach, the same research group analyzed and compared obtained lipid profiles of 28 females with major depressive disorder (MDD), 22 female patients with BD recruited during depressive episodes, and 25 healthy controls [65]. All three groups were age- and BMI-matched. They found similar changes considering lipid classes as in their previous study with BD females (Table 1). In addition, in this study, the researchers found that phSM and CeRG2GNAc1 levels were decreased for the first time. Comparison between MDD and BD groups revealed that levels of LPE and LPI were increased, whereas PE decreased in BD compared to MDD patients. A total of 172 potential biomarkers of BD were identified that could distinguish female BD patients from healthy females (138 upregulated and 34 downregulated in the BD group). A set of eight lipids belonging to four lipid classes, LPE(16:0), LPE (18:0), PC (36:6), PC (38:8), PC (8:0/0:0), PS (42:9), TG (18:0/16:1/22:6), and TG (22:4/17:1/18:2), represents a panel that could be used to distinguish BD patients from healthy controls with moderate reliability. Furthermore, in this study, Zhang et al. (2022) revealed that a biomarker panel of 13 lipids, Cer(d18:1/16:0), Cer(d18:1/24:1), Cer(d40:1), Cer(m18:0/20:0), Cer(m34:0+O), DG(21:5), LPE(18:1), LPE(20:4), PC(16:1/16:0), PE(P-16:0/22:6), PE(P-20:0/18:2), PE(37:2), and PE(8:0p/12:3), could provide reliable discrimination between BD and MDD patients [65]. Among these differential lipids, all Cer was upregulated in BD patients, as well as DG(21:5), LPE (20:4), and PE (8:0p/12:3), while other lipids belonging to the GP class were downregulated.

Recently, Tkachev et al. (2023) [50] published the results of a multicohort study that included patients with SCH, BD, MDD, and healthy controls. Potential biomarkers associated with BD were identified using data from a smaller cohort (36 BD patients from Russia) and from a larger cohort (148 BD patients from Germany and Austria). The study design enables researchers to minimize the influence of factors like diet, lifestyle, and health conditions, including metabolic disorders, on lipid profiles. Plasma lipidomic profiles were analyzed using an untargeted LC-MS method, resulting in the detection of 1361 lipid features and the identification of 395 lipids that belonged to 16 lipid classes. The alterations in lipid profiles of BD patients were broadly shared with those obtained for MDD and SCH in this study. The similarity in lipidome changes across the three psychiatric disorders may be due in part to the overlap in their genetic risk factors, including 20 genes that influence lipid levels out of a total of 84 identified. At the level of lipid classes, downregulation of acyl-CARs and ether-PC, along with the upregulation of Cer, was characteristic for all three diagnoses of SCH, BD, and MDD. Among individual lipid species, 47 molecules were associated with BD in both cohorts, distinguishing patients from healthy controls. However, only 21 of these molecules were identified as specific lipids belonging to the acyl-CAR, Cer, FFA, and SM lipid classes. Changes in individual (di)acyl, alkyl and acylalkyl GPs, LPE, PC, PE, as well as those observed for TGs were mostly observed in a smaller BD cohort but were not reported in a larger BD cohort.

To identify potential diagnostic biomarkers, Costa et al. (2023) compared plasma lipidomic alterations associated with drug-naïve patients with BD (n = 30, both genders) and SCH (n = 30) aged under 60 years with the control group (n = 30) using an untargeted metabolomics approach based on LC-MS [41]. Among the identified differential lipids, 42.88% of lipid species associated with BD belonged to the GP class, while 32.97% belonged to SL class, making them the most significant contributors to the BD discrimination from healthy controls. Average intensities of GLs, such as TGs, and SPs, mostly SMs, were higher in the BD group compared to the control group, as were the intensities of some lipids belonging to the GP, FA, and ST classes. Significant up- or downregulation was identified for 119 lipid compounds. Metabolic pathway analysis of these differential lipids suggested potential effects on the following lipid metabolism pathways: metabolism of androgens and estrogens, FAs (linoleic and arachidonic metabolism), vitamin D3 and globo-series glycosphingolipid and GP metabolism (including PIP), and bile acid and prostaglandin synthesis. Alterations in these metabolic pathways could be associated with a disturbance in key features regulated by specific metabolites. For example, estrogens influence food intake, body weight and fat distribution, glucose homeostasis and insulin sensitivity, inflammation, energy expenditure including the balance between lipolysis and lipogenesis, reproduction, and cognition for estrogens [67]. Similarly, vitamin D plays a role in calcium and phosphate metabolism, cardiovascular health, cell growth and differentiation, and hormonal balance, and both innate and adaptive immune responses [68]. Prostaglandins are primarily involved in immune regulation and resolution [69]. The roles of vitamin D in both depressive and manic episodes, fluctuations of estrogens in the etiology of bipolar disorder, and prostaglandins in rapid cycling in BD have already been explored and confirmed in previous studies [70,71,72].

Jadranin et al. (2023) [66] performed an untargeted LC-MS-based analysis of serum samples of 31 drug-treated patients with BD and 31 control individuals. Blood samples were obtained in the morning after overnight fasting. Among identified lipids, 56 lipid species significantly contributed to the separation between the BD and the control groups. Most of these lipid molecules belonged to following lipid classes, PC (n = 8), ether PC (PC(O-)) (n = 15), SM (n = 10), and TG (n = 12), and 52% of differential lipids were from GP classes. Serum levels of differential lipids were downregulated in BD compared to control individuals, with the exception of Cer (34:1), whose peak intensity was more intensive in the group of BD patients compared to healthy controls. Furthermore, PC (O-34:2), SM (42:1), and CE (18:2) were identified as potential biomarkers of BD with the highest variable importance in projection (VIP) value (higher than 2.0) in a partial least squares-discriminant analysis (PLS-DA) model.

#### Overview of Alterations of Individual Lipid Classes and Species in Patients with BD Compared to Healthy Controls

In this section, a critical analysis of findings from relevant studies on plasma and serum lipidomics in bipolar disorder (Section 3.1) will be provided, highlighting consistent patterns, specific alterations in lipid profiles, and the replication of the findings across studies.

Altered levels of lipid species belonging to the PI and PC classes indicate that GP metabolism could be dysregulated in BD patients compared to healthy controls. Several studies have shown alterations in PI levels in patients with BD [63,73,74]. Molecules from the PI class are involved in the regulation of GP metabolism, including the production of PG, PC, PS, and cardiolipins (CLs) [75]. An increase in PI levels could induce adverse effects on the synthesis of other GP molecules in patients with BD. The increase in the content of the PI fraction was also observed in the study by Guo et al. (2022), but none of the individual PI molecules were identified as a reliable potential biomarker for the disease. Regarding the GP class, PC levels were found to have decreased, and their association with BD was complex, as some molecules were increased while others were decreased [51]. There are only a few plasma lipidomic studies on BD patients that revealed that a set of potential biomarkers could include molecules from the GPs’ major lipid class [52,63,65]. However, there were no overlaps when comparing different studies for PI (40:3), PG (32:4(OH)), PA (48:8(OH)), PA (44:4), PE (42:5), PS (16:1/22:4), PC (36:6), PS (42:9), LPE (16:0), LPE (18:0), PC (38:8), and PC (O-34:2). Additionally, the molecules PC (8:0/6:0), PC (36:6), and PS (42:9) were identified as potential biomarkers in two studies conducted by the same research group [52,65].

Several studies have shown that serum TG levels were elevated in BD patients [62,76,77]. Hiller et al. (2023) reported in their review article on lipid biomarkers in patients with BD that increased serum TG levels may also be a characteristic of the disease [60]. Ribeiro et al. (2017) [63] identified TG (42:3) as a potential biomarker for BD, while Guo et al. (2022) [51] found that plasma levels of TG (16:0/16:1/22:6), TG (16:0/20:4/22:5), TG (22:4/17:1/18:2), and TG (18:0/8:0/20:4) exhibited high reliability as potential biomarkers for BD diagnosis in female patients. In addition, total plasma TG concentrations were not associated with BD [51]; however, the levels of TG (16:0/16:1/22:6), TG (16:0/20:4/22:5), TG (22:4/17:1/18:2), and TG (18:0/8:0/20:4) were significantly associated with the severity of clinical symptoms, as well as factors such as medication use, other diagnoses, and clinical symptoms.

The lipid molecules that are the most promising candidates for biomarkers of BD come from the Cer class. The lipid signaling molecules belong primarily to SLs (including Cer), eicosanoids, and endocannabinoids, which are synthetized from PUFAs and lysophosphatidic acids (LPAs). The metabolic pathways of Cer production in the body are a de novo synthesis by serine palmitoyl transferase and ceramide synthetase, a reacylation of sphingosine by the enzyme sphingosine N-acyltransferase, and a hydrolysis of SM via the action of sphingomyelinases. Some antidepressants and antipsychotics including olanzapine act as inhibitors of acid sphingomyelinase, suggesting that the metabolic transformation of SMs into Cer could be an important factor in mood stabilization and generally in psychosis [78]. Altered acid sphingomyelinase activity and the accumulation of Cer in the rodent brain in animal models of depression induce adverse effects on neurogenesis and neuroprogenitor proliferation [79]. The over-activation of acid sphingomyelinases seems to be associated with oxidative stress in depressive patients [80,81]. Numerous studies have reported alterations of Cer levels in a variety of diseases including diabetes mellitus, obesity, and cardiovascular diseases [81,82,83,84,85]. As the content of Cer is associated with multiple diseases, the analysis of Cer profiles by comprehensive lipidomic analyses is needed to evaluate if levels of some lipid species belonging to the Cer class could be potential biomarkers for BD. Brunkhorst-Kanaan (2019) revealed that serum levels of several Cers were increased in unipolar and bipolar depression patients, and that the use of drugs that are acid sphingomyelinase inhibitors did not lead to their decrease [64]. The changes in SM and Cer levels in depression seem to be complex as the use of drugs lead to weight gain in the patients [86]. Guo et al. (2022) found that Cer levels were increased in females; however, none of the individual lipid molecules from this class were among the nine metabolites identified as a potential set of biomarkers of the disease [51].

In a recent study, Tomasik et al. (2024) prepared an online questionnaire with 635 questions to identify BD patients who were misdiagnosed as MDD patients [87]. The study included 214 patients aged between 18 and 45 years. Their plasma metabolites were analyzed using a targeted MS-based platform, quantifying 630 metabolites belonging to 22 biochemical classes in fasting blood samples. From lipid molecules, molecules from the classes of acyl-CARs, Cers (including hexosyl-, dihexosyl-, trihexosyl-derivatives), cholesterol esters, LPC, PC-acylacyl and alkylacyl, SMs, and TGs were identified. Of the 214 patients with depressive symptoms included in the study, 67 (with a mean age of 28.1 years) were diagnosed with BD. In the validation cohort, which included 30 individuals, 9 (30%) were misdiagnosed, and thus the misdiagnosis rate was similar to the 27.8% found in the study. The metabolomics analysis of plasma revealed that a panel of 17 potential biomarkers could be useful for distinguishing between BD and MDD patients, with Cer(d18:0/20:4) emerging as the strongest predictor for BD classification. In addition to this Cer, eight other metabolites belonging to the lipid class were identified, including hydroxySM (14:1), PC (32:3), hexosyl-Cer(d18:1/22:0), SM (20:2), TG (17:1/36:3), TG (18:1/38:5), PC (36:5), and PC (42:0). The identified biomarkers were primarily associated with the presence of manic episodes during the patients’ lifetime. The findings of this study are particularly important for developing a test for the differential diagnosis of BD from other psychiatric diseases with overlapping symptoms such as MDD and SCH and highlight the potential role of Cer in BD, as well as in mood disorders more broadly.

Finally, only one of the selected seven lipidomic studies of bipolar disorder patients involved drug-naïve patients. Considering that drugs used for the pharmacological treatment of BD, mood stabilizers and antipsychotics, can affect lipid metabolism, their potential impact on lipidomic profiles is an essential consideration. This area warrants further research, as it represents a significant limitation in the current literature.

### 3.2. Serum and Plasma Lipidomics Studies in Patients with SCH

Recently, Messinis et al. (2024) published a comprehensive systematic overview of lipidomic biosignatures in SCH [58]. The authors identified a total of eighteen studies that have employed various methodological approaches to determine lipid profiles of different starting materials (brain, erythrocytes, platelets, urine), with ten of these studies focusing on serum and plasma samples. We chose to include three of these ten studies in our review, as the remaining seven did not meet our inclusion criteria. Specifically, five studies were published more then ten years ago [88,89,90,91,92], four did not utilize LC-MS-based methodologies for determining serum and plasma lipidomic fingerprints [80,81,82,83,84,85,86,87,88,89,90,91,92,93], and one study involved patients at high risk for psychosis rather than SCH patients [94]. Focusing on studies that met our established criteria, this review aims to present the most recent insights into serum and plasma lipidomics profiles determined in SCH patients, determined by the most commonly employed methodological approach (with included studies presented in Table 2) in Section 3.2.

Wang et al. (2019) analyzed serum samples obtained after overnight fasting from 119 patients with SCH and 109 age-, gender-, and ethnicity-matched healthy controls [48]. The patients were under 40 years of age, with 28 of them being first-episode and drug-naïve, while the remaining 91 were in a drug washout period for at least one month. The serum metabolite profiles were determined using an untargeted metabolomics approach based on LC-MS, and a total of 391 features were identified, including phospholipids from the classes PC, LPC, PE, LPE, and SM. Regarding potential lipid biomarkers of SCH, significant alterations in the levels of 51 lipid species were observed (Table 2). In the SCH patients, total levels of LPE and SM were increased, as well as differential lipids from the LPE and SM classes (except for LPE (20:0), which was downregulated). In contrast, total levels and differential lipids from LPC (except for LPC (22:6)) and all PEs belonging to the PE(O-) and PE(P-) subclasses were downregulated compared to the control group. Of the 21 differential lipid species from the PC class, 6 were increased and 15 were decreased, suggesting that the impact of the disorder on lipid metabolism within this class is complex. Among differential PC species, all molecules from the PC(O-) and PC(P-) subclasses were downregulated, and levels of diacyl-PC compounds containing palmitic acid in their structure were upregulated. No significant correlation was detected between differential lipid levels and symptoms. A set of six lipid metabolites, including LPC (18:0), LPC (20:0), PC (18:2/18:2), PC (O-16:0/18:2), LPE (20:4), and PE (P-18:0/18:2), effectively distinguished patients from controls, with good classification performance in the PLS-DA model (AUROC value 0.991).

Using an untargeted lipidomic analysis, Yan et al. (2018) measured levels of 445 serum lipids belonging to 17 classes in overnight fasting plasma samples of 20 hospitalized SCH patients (both pre- (naïve SCH) and post-antipsychotic treatment for eight weeks) and 29 healthy controls [49]. Comparing naïve SCH patients and control individuals, 47 lipid species from 9 lipid classes were significantly dysregulated: cholesterol esters (CE), LPCs, SMs, and TGs were upregulated, while PC(P-)s, PE(P-)s, PCs, LPCs, LPEs, and acyl-CARs were downregulated. In this study, Yan et al. (2018) have revealed several new SCH-markedly dysregulated lipids belonging to SM, acyl-CAR, and Cer classes [49]. The antipsychotic treatment of naïve SCH patients led to a downregulation of 50 lipid species from CE, Cer, FA, GlcCer, LPC, PC, PC(P-), SM, and TG classes. Interestingly, the expression of four regulation patterns was observed: (1) the downregulation of CEs and TGs, lipids that were upregulated before treatment; (2) the further downregulation of PC(P-)s, PCs, and LPCs that were already downregulated in naïve patients; (3) the downregulation of Cers, GlcCers, and FFAs that were not changed in naïve patients; and (4) no effect on the downregulation of PE(P-)s, LPEs, and acyl-CARs that were already downregulated in naïve SCH patients. The first and the fourth patterns could be of great importance in SCH onset or progression of the disease. Based on the results obtained by Yan et al. (2018) [49], potential biomarkers for monitoring therapy would be lipid species belonging to classes of CEs and TGs that were identified to be upregulated in SCH patients before treatment. Additionally, the side effects of the therapy could be associated with therapy-induced increases in lipid species from Cer, GlcCer, and FFA classes.

Cao et al. (2019) analyzed the plasma profiles of 29 individual acyl-CARs in 225 patients with SCH (40 drug-naïve patients with the first episode of psychosis (FEP) and 185 at least one-month drug-free patients) and 175 healthy controls using targeted LC-MS-based analysis [95]. The patients and controls enrolled in the study were age- and sex-matched. Furthermore, besides baseline acyl-CARs levels in a group of 156 patients, their concentration was determined after eight weeks of treatment, which resulted in increased BMI, waist circumference, TGs, VLDL, and decreased fasting blood glucose and HDL levels. At baseline, the concentrations of 11 acyl-CARs were altered, with 2 upregulated (C4-OH, C16:1) and 9 downregulated (C3, C8, C10, C10:1, C10:2, C12, C14-OH, C14:2, and C14:2-OH) in the SCH group compared to the control group. The treatments affected the levels of 13 out of the 29 measured acyl-CARs, and only the levels of C3 and C4-OH were statistically significantly increased. In the post-treatment group, the concentration of 14 acyl-CARs was lower compared to control subjects. Among these decreased acyl-CARs, the levels of C2, C12:1, C14:1, C16:2, C16:2-OH, and C18 were not decreased at baseline. Furthermore, C3 and C12 were decreased at baseline but not after antipsychotic treatment, indicating that therapy could favorably influence their levels. Finally, multivariate analysis showed that plasma levels of C3, C4, and C16 were significantly higher in the recurrent group compared to the FEP before the treatment. After eight weeks of treatment, in recurrent groups beside C3 and C4, the level of C5 was higher compared to the levels obtained in the FEP. Interestingly, it seems that antipsychotic drugs selectively affect the levels of individual acyl-CARs. Based on the results of this study, analysis of serum and plasma profiles of acyl-CAR could be useful for monitoring therapy and investigating the potential association of between therapy side effects with levels of C3, C4, and C16 acyl-CAR in future research.

Leppik et al. (2020) conducted a targeted lipidomic analysis involving 53 patients with a FEP diagnosed with various SCH spectrum disorders [96]. Among these, 44 patients were analyzed both before and after seven months of drug therapy. Thirty-three patients were treated with different antipsychotics, including quetiapine, olanzapine, aripiprazole, risperidone, sertindole, clozapine, and perphenazine, while eleven required additional mood stabilizers, antidepressants, and hypnotics. The lipidomic profiles of these FEP patients (before and after treatment) were compared to those of 37 healthy control subjects. Disease severity was assessed using the BPRS, which showed a reduction after seven months of treatment. Fasting serum samples were analyzed using an LC-MS-based platform, which detected 105 lipid species across three lipid classes: LPCs (n = 14), PCs (n = 76), and SMs (n = 15). Distinctions were also made between ester and ether bonds in GPs. In the FEP group, serum levels of LPC(20:4) were significantly higher, while levels of 16 PCs (including both diacyl-PC (n = 11) and acyl-alkyl PC (n = 5)) and SM(20:2) were significantly lower compared to healthy controls. In addition, the ratio of PC to LPC was lower, whereas the ratio of LPC(20:4) to LPC(20:3) was higher in patients than in controls. The strongest associations with disease onset were observed for the levels of PC(32:2), PC(34:3), PC(34:4), PC(36:2), and PC(36:3). After seven months of treatment, levels of two LPCs (LPC(14:0) and LPC(20:3)) and nine diacyl-PCs increased, while those of two SMs (SM-(OH)-16:1 and SM(18:0)) decreased. This resulted in an increased PC-to-SMs ratio and a decreased LPC(20:4)/LPC(20:3) ratio. The most notable changes were observed for the identified potential biomarkers of SCH spectrum disorders, specifically PC(36:2) and PC(36:3). In treated FEP patients, serum levels of LPC, PC, and SMs became comparable to those of healthy subjects, indicating that drug treatment induced favorable changes in these lipid classes.

Wang et al. (2021) quantified serum levels of 177 metabolites from fatty acids and phospholipid classes in patients with SCH and healthy controls using a targeted LC-MS-based platform [97]. They employed the same patient groups as in their previous study in that they determined serum phospholipid profiles [48]. In SCH patients, lipidomic profiles were significantly altered, and 110 potential biomarkers of the disease were identified by comparing the patient group with healthy controls. Among the identified differential lipids, those containing choline in their structure were the most numerous, including 25 PCs, 23 LPCs, and 11 PC(P-)s). Furthermore, lipidomic changes were observed for individual lipid species across all other identified lipid classes, including six FFAs, seven PEs, nine LPEs, six PE(P-)s, and thirteen SMs. Fold change, calculated as the ratio of median metabolite levels in patients versus healthy controls, was greater than one for all FFAs, 2 LPCs containing PUFA residues (LPC(20:4) and LPC(22:6)), 11 of 25 PCs (including PC(16:0/14:0), PC(16:0/16:0), PC(16:0/18:1), PC(16:0/20:4), PC(16:1/14:0), PC(16:1/18:0), PC(22:5/16:0), PC(22:6/16:0), PC(22:6/18:0), and PC(18:2/16:0)), PC(P-16:0/16:1), most LPEs (including LPE(16:0), LPE(20:4), and LPE(22:6)), five PEs (PE(16:0/18:1), PE(18:0/18:1), PE(20:4/18:0), PE(22:6/16:0), and PE(22:6/16:0)), and six SMs (SM(d18:1/18:0), SM(d18:1/24:1), SM(d17:1/24:1), SM(d18:1/18:1), SM(d18:1/19:0), and SM(d18:2/24:1)), and these 33 lipids were upregulated in SCH patients, although more differential of lipid species were downregulated (n = 77). The changes in lipidomic profiles of PEs, PCs, and SMs were complex, as certain lipid species were increased while others decreased, and the total levels of these lipid classes were comparable between the patient and control groups. Total concentrations of LPC, PC(P-), and PE(P-) decreased. Calculating the product-to-precursor ratios of the metabolites, the desaturation of SFAs to MUFAs appeared to be increased, elongation was weakened, and phospholipase-mediated transformations of PC and PE to their corresponding LPCs and LPEs with SFAs and MUFAs were decreased, whereas those containing PUFAs increased. FFA(17:1), FFA(14:0), FFA(17:0), and FFA(16:1) had the highest variable VIP values in the orthogonal partial least squares discriminant analysis (OPLS-DA) model, and their contribution to group separation was the largest. Among the differentiated lipids, only some PEs correlated with disease symptoms (positive PANSS scores).

Wang et al. (2022) studied plasma samples of 31 non-obese, normotensive SCH antipsychotic-treated SCH patients, 32 age-, sex- and BMI-matched healthy controls, and 35 MDD patients, who all fasted at least 10 h before the sampling [98]. An untargeted lipidomic analysis was performed to measure 782 lipids that belonged to 30 lipid classes. The plasma concentrations of acyl-CARs and PEs decreased, and those of LPCs, LPEs, LPIs, PIPs, and PIP2s increased comparing SCH patients and healthy controls. Obtained lipidomic results correlated with PANSS scores, indicating that identified potential biomarkers of SCH could be both markers of the disorder onset but also of its severity/symptomology. Alterations in 103 lipid species (49 upregulated and 58 downregulated) were observed by comparing serum lipidomics profiles of patients with SCH and those of individuals of the healthy control group. The analysis of these differential lipids revealed that metabolic pathways involved in the endogenous transformation of GPs, linoleic acid, alpha-linolenic acid, and the pathway of glycosylPI (GPI)-anchor biosynthesis could be affected in the SCH group.

As previously mentioned in the section on BD (Section 3.1), Tkachev et al. (2023) performed a multicohort study recruiting patients (436 SCH, 184 BD, and 256 MDD, those with a FEP) and healthy controls (n = 572) from Western Europe (Germany and Austria), China, and Russia [50]. The study design reduces the influence of demographics and environmental variables, including lifestyle and health conditions, by including these three cohorts of SCH patients. The lipidomic signature of patients with SCH was reproducible, and the observed alterations in lipid profiles were not associated with symptomatology. The SCH-associated changes at the levels of lipid classes were observed, including increases in Cers, TGs, and PCs and decreases in acyl-CARs and ether-PCs (Table 2). Of the identified lipids, 77 of 365 significantly differed in the patients compared to healthy controls, and these lipid species belonging to 14 lipid classes could represent potential disease biomarkers. The alterations of lipidomic profiles in SCH patients overlapped with those in BD and MDD patients and this could be at least partly explained by the sharing of genetic factors. Enrichment analysis determined that genetic factors significantly affect the lipid classes of PC, PE, and Cer. A direct comparison of plasma lipidomic signatures of drug-treated and FEP patients showed that antipsychotics could induce changes in profiles of TGs, SMs, PCs, and acyl-CARs. The study conducted by Tkachev et al. (2023) made a significant step forward in establishing clinical diagnostic tests for SCH and other psychiatric disorders, including BD and MDD [50].

In a lipidomics study, Costa et al. (2023) recruited drug-naïve patients with SCH (n = 30) alongside BD patients (n = 30) and healthy controls (n = 30) [41]. Their plasma samples were analyzed using an untargeted lipidomic approach via LC-MS. Generally, the SCH group exhibited lower relative concentrations for most lipid species compared to the control group. When evaluating the lipid species with a higher mean intensity in SCH than in the control group, several GPs such as LPC, particularly LPC16:0, and SPs such as some glycosphingolipids and Cers, as well as several types of STs and FFAs, were prominent. Metabolic pathway analysis indicated that the altered pathways were similar to those identified for the BD group; however, the biosynthesis of arachidonate prostaglandins and vitamin D3 did not appear to be significantly altered in SCH. Conversely, the metabolism of the ganglio series of glycosphingolipids, which did not seem to be affected in the BD group, could be significantly changed in SCH patients.

In an untargeted lipidomic study, Marković et al. (2024) analyzed serum samples from 30 individuals with SCH treated with antipsychotics and 31 non-psychiatric controls using LC-HRMS [99]. A notable feature of this study was the establishment of gender-differentiated SCH and control groups. The abundances of the majority of differential lipids were downregulated in SCH patients compared to controls, both in females (60 lipid species belonging to LPC, FFA, Cer, PC, PC(O-), SM, DG, and TG classes) and males (49 species belonging to LPC, FFA, Cer, PC, PC(O-), SM, DG, TG, and CE classes). Specifically, Cer (34:2), Cer (34:1), LPC (16:0), and TG (48:2) were four identified lipids with higher levels in both SCH female and male patients compared to their respective control groups. In many studies, researchers found increased Cer levels in patients with BD, but only a few studies of SCH patients observed that plasma or serum Cers were elevated. One of those studies was a recent multicenter study with a large number of patients with SCH performed by Tkachev et al. (2023) [50]. Furthermore, according to the results obtained, Markovic et al. (2024) hypothesize that SCH-associated alterations in lipidomics may be strongly associated with increased phospholipase A2 (PLA2) activity [99].

Shi et al. (2024) performed untargeted ultrahigh performance LC-MS/MS lipidomics analysis in 96 SCH male patients and 96 healthy men [100]. In serum samples collected after overnight fasting, 1033 metabolites were detected, including 115 FFAs, 299 GLs, 472 GPs, 114 SLs, 30 STs, and 3 isopentenols. The authors reported that of 34 differential lipids, 21 were downregulated and 13 were upregulated in SCH patients compared to controls. Most of the metabolites being identified as potential biomarkers of SCH were from the GP category (21 lipid species including 2 PA, 3 PC, LPC (22:6), PE (16:1/18:0), 9 ether-PE of alkyl (6 PE(O-)), and 3 alkenyl(PE(P-)), LPE (20:3), 3 PI, and 1 phosphatidylmethanol (PMeOH) (PMeOH(16:0/18:1)). Eight GPs were significantly upregulated (all three wer lipids that belonged to the PI class, PC (16:0/18:1), PA (16:0/16:0), LPE (20:3), PE (16:1/18:0), and PMeOH (16:0/18:1), and thirteen were downregulated including all nine ether-PE lipid species. Other factors that were associated with SCH were age, BMI, smoking, and educational level. Furthermore, the causal relationship between lipidomic profile and cognitive performance in five domains (immediate memory, visuospatial/constructional, language, attention, and delayed memory) was revealed for ten lipid species, and among them, taurochenodeoxycholic acid and PE (O-17:1/22:4) were significantly associated with a total cognition score (total RBANS score).

#### 3.2.1. Overview of Alterations in Individual Lipid Classes and Species in Patients with SCH Compared to Healthy Controls

In the last ten years, several papers on plasma and serum lipidomics LC-MS-based studies of SCH patients have been published. Altered levels of acyl-CARs [33,34,84,87] and ether-PLs (including plasmalogens) [48,49,50,95,98,99,100] have been suggested as potential biomarkers of SCH in numerous studies. At the same time, the metabolism of GPs has been found to be dysregulated in SCH patients and is associated with changes in the levels of the PC lipid class, as well as the content of individual lipid species from this fraction [48,49,96,99]. Additionally, changes in the levels of LPC, LPE, PE, SM, TG, and CE lipid moieties have often been identified in lipidomic studies over the last decade [41,48,49,50,96,97,98,99,100].

#### Alterations in Serum and Plasma Profiles of Acyl-Carnitines in SCH Patients

During β-oxidation, CARs are responsible for moving long-chain FAs across the inner mitochondrial membrane [101]. Their action also influences the redox status, the activity of certain enzymes, and it heightens cholinergic neurotransmission [102]. CARs can be provided from diet, with rich sources being meat and dairy products, or synthesized in the liver and kidneys from L-lysine and L-methionine [103]. According to the literature of available data, acyl-CAR imbalance exists in neuropsychiatric disorders, including SCH, and may primarily induce bioenergetic abnormalities, along with changes in insulin sensitivity, inflammatory response, and lipid metabolism [102,104,105]. Unexpectedly, some studies showed harmful effects of acyl-CARs on insulin action that were associated with the induction of mitochondrial and oxidative stress [106]. Analysis of acyl-CARs serum profiles, as reported by Cao et al. (2019), indicated a decrease in the levels of medium- and long-chain acyl-CARs (C6-C18) in SCH patients, with a correlation observed among their levels [95]. A further reduction in their levels was observed following the drug treatment. This finding was in line with previous studies suggesting that some antipsychotics or mood stabilizers can induce a secondary deficiency in CARs, which in turn leads to an acyl-CAR deficiency [107]. It is worth noting that drug treatment led to an increase in short-chain C3 and C4-CARs [95], implying that the activity of carnitine palmitoyltransferase 1, carnitine acetyltransferase, or/and enzymes related with acyl (C3 and C4)-CoA could be dysregulated. The acyl-CAR was also dysregulated in other diseases like type 2 diabetes [108] which may be relevant when evaluating the potential of these molecules as biomarkers for neuropsychiatric disorders including SCH. Several other lipidomics studies in the last ten years have also identified alterations in acyl-CARs levels [49,50,95,98]. In the study conducted by Tkachev et al. (2023), plasma levels of 12 acyl-CARs lipid species were found to be downregulated compared to healthy controls, but similar changes were characteristic for BD patients, too [50]. Certainly, improving the acyl-CAR status could have merit, and the use of acyl-CAR dietary supplements in SCH patients should be considered as a possible treatment approach [109].

#### Alterations in Serum and Plasma Glycerophospholipid Profiles in SCH Patients

According to the available data in the literature, GPs could play a crucial role in SCH pathogenesis [110]. Alterations in PC and LPC levels could be closely related to the activities of lecithin-cholesterol acyltransferases (LCATs) and phospholipases A that can transform PC to corresponding LPC [111,112]. Several lipidomic studies in the last ten years have reported a significant decrease in the total serum levels of the LPC fraction [48,49,97]. These changes could be associated with a decrease in HDL levels and could also reflect the decreased synthesis of lipid mediators from PUFAs [88,89]. Considering lipid species within this class, LPC molecules containing SFAs and MUFAs may differ from those containing PUFAs, as LPC molecules originate from multiple metabolic pathways. Blood LPC can be synthesized in the liver or directly in the bloodstream from lipoprotein-derived PCs [111,112]. This process involves the action of LCAT and/or one or more phospholipases A, resulting in the production of LPCs with SFAs in their structure. It is also worth noting that a decrease in LPC levels might be associated with a decrease in the serum levels of its precursor serum PCs. At the same time, LPC could possess both pro- and anti-inflammatory properties [113], and inflammation is closely associated with neurodegeneration, but also inflammatory disorders. For individual LPCs, a potential biomarker could be LPCs with SFAs like LPC(18:0) and LPC(20:0), that were found to be decreased, as well as LPC(22:6) and LPC(22:4) that were found to be increased in SCH.

Numerous studies have documented changes in PC levels, a group of GPs containing a choline headgroup that could be accompanied with Pes’ decline within the brain tissue, blood, plasma, or serum of individuals with SCH [50,61]. The results regarding the decrease in plasmalogens, complex GPs that contain a vinyl ether link at the sn-1 position, are the most consistent across all lipidomic studies of SCH patients [50,93,97,100]. These molecules contain either PE or PC at the sn-3 position and are enriched with PUFAs, especially arachidonic acid and DHA [103,114]. Plasmalogens are known to be involved in various biological functions and are particularly abundant in the brain, where they participate in the prevention of neuroinflammation, the regulation of cognitive function, and the inhibition of neural cell death [114,115]. Their anti-inflammatory and antioxidant properties are important factors associated with their function in biological systems [116,117]. In a study conducted before 2014, and not using an LC-MS platform for the determination of plasmalogens, Kaddurah-Daouk et al. (2012) observed a decrease in plasmalogens ranging from 10 to 40% and suggested that the decline was associated with oxidative stress [91]. In the last decade, the results for the alteration of plasmalogen levels were confirmed in all studies that were measuring serum levels of this class of lipid molecules [48,49,50,95,98,99,100]. There are two pools of plasmalogens: those synthesized in the liver and gastrointestinal tract, and those synthesized by circulating platelets, and the dynamics of plasmalogens seem to be altered due to changes in lipid transport and remodeling [114]. Beside changes in plasmalogens, Wang et al. (2021) found that PCs with fatty acids 18:0, 18:2, and 20:4 were reduced, and those with a 16:0 fatty acid increased as well as some with 22:5 and 22:6 fatty acids in SCH patients compared to controls [97]. Considering individual PCs, several PCs were identified as potential biomarkers of SCH in several studies, including PC(18:2/18:2) and PC(O-16:0/18:2) [32,33,74]. According to Lepikk et al. (2020), PC(32:2), PC(34:3), PC(34:4), PC(36:2), and PC(36:3) were the most significant contributors to differentiating FEP patients from controls [96]. Furthermore, after seven months of antipsychotic treatment, the levels of these metabolites in FEP patients were found not to be significantly different from those observed in the control groups. The authors suggested that the decline of these PC lipid species could be related to inflammation and the increased transfer of C16, C16:1, C18:1, and C18:2 to CARs, as well as the increased LCAT and PLA2 activities leading to the formation of LPCs [96]. The evidence from several studies supports this hypothesis, including findings that increasing circulating acyl-CAR with 18:2 is accompanied by a reduction in PCs with an 18:2 acyl chain [118,119]. The observed decrease in PE levels in combination with the increased LPE could also indicate that enzyme PLA2 is activated in SCH [87], and the synthesized lysophospholipids could unfavorably influence microglia activation, leading to local inflammation and neurotoxicity [119,120]. At the same time, the activation of phospholipase D induced by chronic stress leading to the hypothalamic–pituitary–adrenal axis could cause the increased transformation of PE and PC to PA, and subsequently to an increase in DGs [99,121]. The decrease in plasma acyl-CARs and PE could be linked to mitochondrial dysfunction in SCH, as these lipids are essential for the transport of FAs across the inner mitochondrial membrane and mitochondrial morphology [101,122]. Finally, there are two main pathways in the de novo biosynthesis of PC [123], which could be involved in the regulation of PC levels in serum and plasma in SCH. The major pathway is the cytidine diphosphate-choline (CDP-choline) pathway (Kennedy pathway), which occurs in all cells with a nucleus, while a minor pathway occurs in liver. The choline is a substrate in the first reaction and is required for the major pathway. The previous NMR-based studies of our research group identified choline as a potential biomarker of both BD [124] and SCH [125], supporting the assumption that PC synthesis de novo could be dysregulated in both these psychiatric diagnoses, too. The increase in PE accompanied by the decrease in corresponding PC could indicate that the second minor pathway that occurs only in the liver is altered. Currently, the ratios of serum PE and corresponding PC species are not analyzed in detail in SCH. So, to explore the potential alterations and their importance in SCH, the levels of PEs and PCs should be quantified in further studies together with the determination of the status of folates and vitamin B12, as they are important co-factors of all methylation reactions of the minor pathway.

#### Alterations of Serum and Plasma Triacylglycerol Profiles in SCH Patients

In numerous previous studies, SCH patients had significantly altered levels and compositions of plasma and serum TGs which contained higher levels of lipid species with long-chain FAs and SFAs, compared to controls [58,88,89]. In two previous studies by Orešić et al. (2011, 2012), the content of TGs with saturated fatty acids and the most abundant TG lipid species were increased, but those with PUFA were not [88,89]. The authors also revealed that these changes were in correlation with insulin sensitivity and BMI. At the level of lipid species, the amounts of serum TG(16:0/18:1/18:1) and TG(18:2/18:2/18:1) were associated with brain grain density [88]. Summarizing serum/plasma lipidomic studies in the last ten years, Tkachev et al. (2023) have found that 21 TG lipid species were dysregulated in SCH [50], Marković et al. (2024) identified that TG(48:2) was upregulated in both group of female and male patients compared to controls [99], and Shi et al. (2024) reported that TG (15:0/16:1/16:1) was significantly increased, and levels of TG (18:1/18:2/22:2), TG (18:2/18:3/22:6), TG (18:2/20:4/20:4), and TG (18:2/20:4/20:5) decreased in SCH patients [100]. The treatment with antipsychotics could additionally affect TG concentrations [126,127,128], and serum/plasma TG profiles. Tkachev et al. (2021) observed that the most-affected TGs by drug treatment (40 different medications) were short-chain TGs (40–48 carbons in FA residues) and not those that were the most abundant in the circulation [126].

#### Alterations in Serum and Plasma Sphingolipids Profiles in SCH Patients

The data regarding the metabolomic profiling of SMs are still quite limited, but Cers and SMs are strongly associated with components of metabolic syndrome, especially concerning the dysregulation of SMs with unsaturated FAs in their structure [129,130]. At the same time, the SMs inhibit the activity of LCAT enzymes, and thus LPC and SM levels could be inversely related [131]. In several recent studies, it was also observed that plasma and serum levels of Cer were upregulated in SCH patients, including the multicentric study conducted by Tkachev et al. (2023) on 436 patients [50]. Research regarding changes in both GPs and SMs in neuropsychiatric disorders deserves strong attention in the future due to their shared role in the structure of the membrane, the transfer of phosphocholine from PCs to a Cer during the synthesis of SM, and the lipotoxicity-induced metabolic stress due to the imbalance of PCs and SMs [132,133,134].

### 3.3. Comparison of Serum and Plasma Lipidomic Fingerprints of Bipolar Disorder and Schizophrenia Patients

According to a systematic literature review, there are only four studies that have concurrently analyzed lipidomic profiles of plasma and serum in both BD and SCH patients [41,50,135,136]. In two of these studies [135,136], the comparisons between the patient groups and the control group are not analyzed as one of the main findings of these studies, and so they were not discussed in previous sections. Recent comparative analyses of lipid profiles in samples from patients with unipolar and bipolar depression have demonstrated that these two conditions can be distinguished based on the content of lipid species of the Cer fraction [87]. In most lipidomic studies involving patients with SCH, no significant differences were found in serum and plasma Cer contents compared to the control group. However, in the recent studies by Tkachev et al. (2023) and Marković et al. (2024), the authors observed that levels of several Cers were elevated in the circulation of SCH patients compared to control subjects [50,99]. Alterations in levels of ceramides in plasma and serum have been found in previous studies including major psychiatric disorders beside BD and SCH such as depression (increased levels of Cer (16:0), Cer (18:0), Cer (20:0), Cer (22:0), Cer (24:0), Cer (24:1), Cer (26:1), hexosylCer, LacCer, and sulfatides), anxiety (increased levels of Cer (16:0), Cer (16:1), Cer (18:1), Cer (23:1), Cer (20:0), Cer (22:1), Cer (24:0), Cer (26:1), and Cer (36:4)), and attention deficit hyperactivity disorder (increased levels of Cer (22:0); decreased levers of Cer (24:0) and Cer (d24:1)) [137]. The discovery of diagnosis-specific lipid biomarkers for SCH and BD cannot be achieved without further lipidomics studies. These studies should include patients with other psychiatric diseases, involve a larger number of participants, and consider important genetic, environmental, and lifestyle factors, and factors associated with these conditions such as insulin sensitivity, metabolic health, weight status, therapy-induced change, dietary habits, substance use (drugs, alcohol, cannabis, and other), gut microbiome composition, and ethnicity. At the same time, several important methodological limitations need to be addressed concurrently to ensure the reproducibility of the measurements, as well as the sensitivity and specificity of the analytical procedure and standardize it, as mentioned earlier in the introduction section.

Tao et al. (2022) [135] performed an analysis of lipidomic profiles of 112 SCH patients, 132 BD patients, 105 patients with MDD, and 198 age-, sex-, BMI-matched healthy controls. The authors did not focus on finding differences between the patients and heathy controls, but on identifying the psychosis subtypes and classifying patients with different diagnoses into these groups. All SCH patients were experiencing a first episode of psychosis and were drug-naïve, as well as 63 of 132 BD patients, and 77 of 105 MDD patients that participated in the study. The patients who were not drug-naïve had at least a two-week drug-wash-out period. Plasma lipid profiles were determined based on UHPLC-MS analysis that allowed the identification of 1164 lipid molecules, and 10 of these lipids were found to be primary discriminators for distinguishing psychotic patients from control subjects. Of these ten psychosis biomarkers, seven were upregulated including fatty aldehyde 9,12-octadecadienoic, diacylglycerol-*N*,*N*,*N*-trimethyl homocysteine, fatty acids-cyclopentane octanoic acid, 12-tridecynoic acid, caprylic acid and hexadecandioic acid, and oxidized PC (16:0/18:1), whereas the vitamin D3-derivate 20-oxo-22,23,24,25,26,27-hexanorvitamin D3, 10-nitro-octadecadienoic acid, and gamma-butyric acid analog 4-amino-3methylbutanoic acid were downregulated. Patients were classified into two subtypes (Cluster 1 and Cluster 2) based on the content of these ten lipid metabolites that represent potential biomarkers of psychotic disorders. The patients classified into the Cluster 2 subtype had lower global assessment scores and showed significant alterations in white brain matter measured by fractional anisotropy and radial diffusivity compared to patients of the Cluster 1 subtype. The SCH (66% of patients) and BD (48% of patients) patients classified into Cluster 2 showed similar biological patterns, indicating that serum and plasma lipid biomarkers could be used to identify transdiagnostic subtypes across psychiatric diseases. It is noteworthy that these ten potential biomarkers have not been measured and identified in any other lipidomics study performed, so far. Among these markers, some could be associated with redox status, while others even could be linked to unhealthy dietary habits (primary marker 20-oxo-22,23,24,25,26,27-hexanorvitamin D3). The findings of this study suggest that further untargeted lipidomics approach studies may be necessary to identify all potential biomarkers of psychiatric diseases including mood psychosis ones.

Numerous studies have provided evidence of a strong link between plasma lipid profiles and psychiatric disorders [28,29,32,138]. Yu et al. (2024) [136] used plasma lipidomics data from a population-based association study (GWAS) involving 7145 Finnish individuals, identifying 179 lipid species across 13 lipid classes [139], to assess the association of five psychiatric disorders (SCH, BD, depression, autism, and anxiety disorder) with plasma lipid profiles. In patients with BD, a strong correlation was found between several PC lipid species (PC (17:0/20:4), PC (18:0/20:4), PC (16:0/20:4), PC (20:4/0:0), PC (16:0/22:5), PC (18:1/20:4), PC (18:2/18:2), PC (18:1/20:2), PC (16:0/20:2), PC (17:0/18:2), PC (18:1/18:2), and PC (16:0/18:2)), two STs (sterol ester (27:1/20:4), and sterol ester (27:1/20:5)) with the risk of the disease. Some PC species, particularly those with a fatty acid 20:4, showed a protective effect, while those with a fatty acid 18:2 were linked to a higher risk of BD. These findings suggest that PC plays a complex role in BD. Additionally, reverse Mendelian randomization analysis showed associations with other lipid species, including PC (14:0/16:0), PC (15:0/18:1), PC (O-16:0/22:5), PC (O-18:2/20:4), PE (16:0/18:2), PE (18:1/18:1), and PI (16:0/18:1). Only the results for plasma levels of PC (18:1/20:2) were derived using both statistical approaches. Considering SCH patients, Yu et al. (2024) observed that levels of 22 lipid species were associated with SCH diagnosis [136]. These lipid species mostly belonged to GP classes (1 PE, 15 PCs, 1 ether-PC, 2 PIs). In reverse Mendelian randomized analysis, association with SCH was shown for levels of three lipid molecules, PI (18:1/20:4), PC (17:0/18:2), and SM (d34:2). However, all obtained results were no longer statistically significant after false discovery rate (FDR) correction, and their association with SCH onset should be assessed and confirmed in further studies. Results of these studies emphasize the significance of the alteration of PC lipid fraction in both BD and SCH, pointing out that the effects could be even more pronounced in BD.

As already described in Section 3.1 and Section 3.2, Tkachev et al. (2023) suggested that SCH- and BD-associated changes in plasma lipidomic profiles are largely shared [50], and one of the main novel findings of the study was that alterations in Cer levels were characteristic for both BD and SCH patients. However, comparing results for Cer profiles, Cer (d32:1) and Cer (d38:1) levels were dysregulated in BD (considering both BD cohorts), but not in SCH patients (considering results of three cohorts involving SCH patients) compared to healthy controls. Moreover, the changes in serum contents of Cer (d43:3) were related to SCH. Furthermore, there were no differences in choline and ethanolamine-containing GPs and TGs when considering both cohorts of patients with BD, suggesting that changes in these lipid classes may be more pronounced in those with SCH. Furthermore, there were no differences for choline and ethanolamine containing GPs, suggesting that changes in these lipid classes could be more pronounced in patients with SCH.

In the study by Costa et al. (2023), the comparison of the lipidomics profiles of BD and SCH revealed that most of the differential lipids were SPs with a percentage of 54.17%, followed by GLs and GPs at equal proportions of 16.67%, and STs and FFAs presented with the lowest number of species [41]. Based on the results obtained, lipidomics analysis using the same analytical approach as that of Costa et al. (2023) [41] for determination of serum or plasma lipid profiles focusing on results for SPs, GLs, and GPs should be validated and could potentially serve as a clinical tool to distinguish between BD and SCH, two diagnoses with overlapping symptoms. This approach may be used as a screening tool or as a complementary test to the standard diagnostic procedure. Most of differential lipids were downregulated in SCH compared to BD patients but also compared to healthy controls. The 28 putatively identified lipid features representing potential biomarkers of BD and SCH diagnosis belonged to 11 different lipid classes, and included the following lipid species: sterol lipid (29:1), Cer (42:2), Cer (44:2), SM (40:1), SM (41:1), SM (42:1), HexCer (42:1), TG (51:7), TG (53:7), TG (54:7), PC (O-44:3), PC (O-44:5), PC (46:7), LPC (16:1), acyl-CAR (10:2), cerebroside 3-sulfate, and a vitamin D3 derivate. When comparing the BD and SCH groups, the metabolism alterations of GPs, glycoshingolipids, linoleic, and arachidonic pathways could differ between the diagnoses, and contribute to their separation and potentially to the discrimination between the groups in relation to lipid metabolism.

In summary, it appears that some alterations in lipid metabolism are shared between both diagnoses; however, further studies could confirm some differences, particularly regarding lipid molecules from the SL lipid class.

## 4. Conclusions

Serum and plasma lipidomics studies of bipolar disorder and schizophrenia showed interrelations between PCs and LPCs, and PEs and LPEs, along with Cers, SMs, and PCs, and indicate that the activities of phospholipases, LCAT, and sphingomyelin synthase could be altered in these disorders, representing potential targets for new drug treatments. Potential lipid biomarkers of these diseases could belong to different serum/plasma lipid classes: fatty acyls (non-esterified fatty acids and acyl-carnitines), glycerolipids (diacylglycerols and triacylglycerols), glycerophospholipids (phosphatidylcholines, lysophosphatidylcholines, phosphatidylethanolamines, lysophosphatidylethanolamines, phosphatidylserines, phosphatidylinositols, and lysophosphatidylinositols), sphingolipids (sphingomyelins, ceramides and their hexosyl derivatives), and sterol lipids (cholesterol esters).

However, there are still several existing gaps, including (1) the demand for a fast, accurate, sensitive, and specific instrumental method for a clinical diagnostic of these psychiatric disorders; (2) the request for the monitoring of the patients’ responses to medical treatments; and (3) the fact that the insufficient understanding of the changes in metabolic pathways altered by diseases are not fulfilled yet. The establishment of these diseases’ universal biomarker sets, based on their blood lipidomics and metabolomics fingerprints, is still missing and is a necessary step. At this moment, universal diagnosis-specific biomarkers remain unconfirmed due to the limited number of analyzed blood samples of patients with adequate metadata, origins, and similar analyses using this methodological approach.

## Figures and Tables

**Table 1:** Serum and plasma lipidomics studies of bipolar disorder.

Reference	Analytical Tool	Number of Participants	Female (%)	Mean Age *	Lipid Classes	Main Findings—Individual Lipids	Main Findings—Lipid Classes
Ribeiro et al. (2017) [63]	UHPLC-MS	14 BD 21 HC	71 67	46 (9) 34 (13)	untargeted analysis, GL, GP, SP, ST, FFA	121 differential lipids, a panel of potential biomarkers for distinguishing BD from HC: PI (40:3), PG (32:4(OH)), PA (48:8(OH)), PA (44:4), PE (42:5), and TG (42:3)	GL ↑, SP ↑, GP ↓ in BD compared to HC, lipid class PI was the most altered
Brunkhorst-Kanaan et al. (2019) [64]	UHPL-MS	67 BD and MDD HC 405	46 65	not reported	targeted analysis: Cer, LPAs, endocannabinoids and pterins; untargeted, 220 lipids, TG, DG, CE, LPC, PI, PC, PE, FFA	C16Cer, C18Cer, C20Cer, C22Cer, C24Cer C24:1Cer; C24:1GluCer, C24LacCer	differences between unipolar or BP patients and the control group originate from ceramides and their hexosyl-metabolites
Guo et al. (2022) [51]	UHPLC-MS/MS	24 BD 30 HC	women	32.5(27.00, 41.5) 29.00(25.00, 35.2)	untargeted, 884 lipids, 31 lipid classes	55 differential lipids, a panel of potential biomarkers for distinguishing BD from HC: PS (42:9), DG (21:5), PC (36:6), PC (8:0/6:0), PS (16:1/22:4), TG (16:0/16:1/22:6), TG (16:0/20:4/22:5), TG (22:4/17:1/18:2), TG (18:0/8:0/20:4, 56:4)	WE ↓, acyl-CAR ↓, SM ↓, coenzyme Q10 ↓, MGDG ↓, PS ↓, PE ↓, PI ↑, LPC ↑, LPE ↑, LPI ↑, PIP ↑, PIP ↑2, Cer ↑, CerP ↑, GD2 ↑, GM2 ↑, TG ↑, MG ↑ in BD patients compared to HC
Zhang et al. (2022) [65]	UHPLC-MS/MS	28 MDD 22 BD HC 25	women	36.5 (30.25, 43.50) 34.00 (27.00, 42.0) 31.00 (26.50, 38.0)	untargeted, 884 lipids, 31 lipid classes	172 differential lipids, a panel of potential biomarkers for distinguishing BD from HC: PC (36:6), PS (42:9), LPE (16:0), LPE (18:0), PC (38:8), PC (8:0/6:0), TG (22:4/17:1/18:2), TG (16:0/16:1/22:6)	WE ↓, acyl-CAR ↓, SM ↓, coenzyme Q10 ↓, MGDG ↓, PS ↓, PE ↓, phytosphingomyelin ↓, LPE ↑, PIP ↑, PIP2 ↑, GD2 ↑, GM2 ↑, TG ↑ in BD patients compared to HC
Tkachev et al. (2023) [50]	UHPLC-MS	Ger/Aus cohort BD 148 HC 187 Russian cohort BD 36 HC 138	48 57 67 22	43.3 (13.4) 38.3 (16.2) 28.2 (9.1) 29.5 (8.3)	untargeted, 1361 features, 395 lipids, CAR, Cer, CE, DG, FFA, LPC, LPC(O-), LPC(P-), LPE, PC, PC(O-), PC(P-), PE, PE(P-), SM, TG	47 differential features, 21 identified lipid molecules: CAR (10:1), (12:2), (13:1), Cer(d32:1), (d34:1), (d36:1), (d36:2), (d38:1), (d40:2), (d40:3), (d42:3), (d43:2), FFA (10:2), (12:3), (13:1), (23:0), (23:1), (25:1), (25:3),(8:0), SM(d36:1)	the characteristic acyl-CAR ↓, and PC(O-) ↓ and Cer ↑ shared by SCH, BD and MDD
Costa et al. (2023) [41]	UHPLC-MS	SCH 30 BD 30 HC 30	46.6 63.3 50.0	26.5 (6.8) 26.6 (4.4) 26.5 (2.2)	untargeted, FFA, LPE, ST, LPS, PC, LPC, LPC(O-), LPA, LPS(O-), PE, PA, Cer, CE, SM, LPI, PA(O-), DG, PG, TG, acyl-CAR, PI(O-), PC(O-), PS	119 differential lipids	most of the identified differential lipids were downregulated compared to control, included lipid classes of FFA, LPE, ST, LPS, PC, LPC, LPC(O-), LPA, LPS, LPI, Cer, CE, SM, PA, TG, DG, PE, PI(O-), PC(O-), PS, PI, PI(O-), PG(O-), acyl-CAR
Jadranin et al. (2024) [66]	LC-HRMS	BD 31 HC 31	58 48.4	not reported	untargeted, 201 features, LPC, FFA, PA, LPS, Cer, SM, PC, PC(O-), PS, DG, TG, CE	56 differential lipids, 55 ↓, and Cer34:1 ↑ in BD The highest VIP scores PC (O-34:2), SM (42:1), and CE (18:2)	differential lipids belonged mostly to GP, and SP main lipid classes, and SM, PC(O-), PC, and TG sub-classes of main serum lipid classes

Mean ages * were presented as average value(stdev) or median value(1st, 3rd quartile); LC—liquid chromatography; MS—mass spectrometry; HPLC—high-pressure LC; UHPLC—ultra-HPLC; MS/MS—MS coupled with MS (tandem MS); BD—bipolar disorder; HC—healthy controls; SCH—schizophrenia; MDD—major depression disorder; acyl-CAR—acyl-carnitines; CAR—carnitine; CE—cholesterol ester; Cer—ceramide; CerP—ceramide-phosphates; DG—diacylglicerols; FFA—free fatty acids; GD2—ganglioside GD2; GL—glycerolipids; GluCer—glucosyl-Cer; GM2—ganglioside GM2; GP—glycerophospholipids; LacCer—lactosyl-Cer; LPA—lysophosphatidic acid; LPC—lysophosphatidyl-choline; LPC(O-)—ether-LPC; LPC(P-)—LPC-plasmalogen; LPE—lysophosphatidyl-ethanolamine; LPI—lysophosphatidyl-inositol; LPS(O-)—ether-LPS; MG—monoacylglycerols; MGDG—monogalactosyldiacylglycerols; LPS—lysophosphatidyl-serine; PA—phosphatidic acid; PA(O-)—ether-PA; PC—phosphatidyl-choline; PC(O-)—ether-PC; PC(P-)—PC plasmalogen; PE—phosphatidyl-ethanolamine; PE(P-)—PE plasmalogen; PG—phosphatidyl-glycerol; PG(O-)—ether-PG; PI—phosphatidyl-inositol; PI(O-)—ether-PI; PIP—phospho-phosphatidyl-inositol; PIP2—bisphospho-phosphatidyl-inositol; PS—phosphatidyl-serine; SM—sphingomyelin; SP—sphingolipids; ST—sterol lipids; TG—triacylglicerols; WE—wax-esters; ↑—upregulated; ↓—downregulated.

**Table 2:** Serum and plasma lipidomics studies of schizophrenia patients.

Reference	Analytical Tool	Number of Participants	Female	Mean Age *	Lipid Classes	Main Findings—Individual Lipids	Main Findings—Lipid Classes
Wang et al. (2019) [48]	UPLC-MS/MS	119 SCH training set 84 testing set 35 109 HC training set 77 testing set 32	66 66	29.13 (6.05) 27.93 (6.67) 30.61 (4.87) 30.5 (3.94)	untargeted metabolomics, 391 features, PC, LPC, PE, LPE, SM	51 differential lipids, a biomarker panel LPC (18:0), LPC (20:0), PC (18:2/18:2), PC (O-16:0/18:2), LPE (20:4), and PE (P-18:0/18:2)	151 differential metabolites/51 differential lipids, total PC ↔, LPC ↓, PE ↓, LPE ↑, and SM ↑
Yan et al. (2018) [49]	LC-DIA-MS	20 SCH 29 HC	45 38	32 (9) 32 (8)	untargeted, 445 lipids, 17 lipid classes	47 differential lipids, antipsychotic treatment downregulated 50 lipid species	CE ↑, LPC ↑, SM ↑, TG ↑, p-PC ↓, p-PE ↓, PC ↓, LPC ↓, LPE ↓, acyl-CAR ↓, antipsychotic treatment downregulated CE, Cer, FFA, GlcCer, LPC, PC, p-PC, SM, TG
Cao et al. (2019) [95]	UHPLC-MS	225 SCH 175 HC	60 69.1	37.31 (10.85) 39.44 (9.36)	targeted, 29 acyl-CAR	C4-OH ↑, C16:1 ↑, C3 ↓, C8 ↓, C10 ↓, C10:1 ↓, C10:2 ↓, C12 ↓, C14-OH ↓, C14:2 ↓, and C14:2-OH ↓	
Leppik et al. (2020) [96]	FIA-MS/MS and LC-MS/MS	53 FEP 37 HC	39.6 56.8	26.2 (6.0) 24.8 (5.3)	targeted analysis, 105 lipids, PC, LPC, SM	differential lipids LPC (20:4) ↑, 16 PCs (11aa-, 5ae-PC) ↓, SM (20:2) ↓ the strongest effect size PC-aa-molecules: PC (32:2), PC (34:3), PC (34:4), PC (36:2), and PC (36:3)	PC/LPC ↓ in SCH compared to HC
Wang et al. (2021) [97]	UPLC-MS	119 SCH 109HC	56.3 66.1	29.0 (25.0, 33.3) 30.0 (26.0, 33.0)	177 lipids, FFA, GP classes	110 lipids upregulated 5 of 6 FFA (decreased FFA20:4), 2 of 23 LPC, 11 of 25 PC, 3 of 9 LPE, 5 of 7 PE, most lipids were downregulated, highest VIP values FFA (17:1), FFA (14:0), FFA (17:0), FFA (16:1)	LPC ↓, PC(P-) ↓, PE(P-) ↓ in SCH compared to HC
Wang et al. (2022) [98]	LC-MS	SCH 31 MDD 35 HC 32	59.3 65.7 68.5	28 (24, 37) 34.5 (28.5, 38) 29 (25, 36.75)	untargeted lipidomics, 782 lipids, 30 lipid classes	105 differential lipids 45 ↑, 58 ↓ 50 PC lipid species	acyl-CAR ↓, PE ↓, LPC ↑, LPE ↑, LPI ↑, PIP ↑, PIP2 ↑ in SCH compared to HC
Tkachev et al. (2023) [50]	UHPLC-MS	Chinese SCH 170 HC 153 Ger/Aus SCH 184 HC 187 Russian SCH 82 HC 138	53 54 30 57 23 22	36.9 (11.6) 37.8 (11.3) 39.2 (12.8) 38.3 (16.2) 31.2 (8.4) 29.5 (8.3)	1361 features, 395 identified lipid species, CAR, Cer, CE, DG, FFA, LPC, LPC(O-), LPC(P-), LPE, PC, PC(O-), PC(P-), PE, PE(P-), SM, TG	12 acyl-CAR, CE (22:5), 10 Cer, DAG (36:2), FFA12:2, FFA13:1, LPC (22:4), LPC (O-26:1), nine PC, PC (O-34:0), six PC(P-), three PE, four PE(P-), five SM, 21 TG	the characteristic acyl-CAR ↓, and ether-PC ↓, and Cer ↑ shared by SCH, BD, and MDD
Costa et al. (2023) [41]	UHPLC-MS	SCH 30 BD 30 HC 30	46.6 63.3 50.0	26.5 (6.8) 26.6 (4.4) 26.5 (2.2)	all main lipid classes GP, GL, SP, ST, FFA	49 lipid species	most of the identified differential lipids were downregulated (↓) compared to controls, belonged to lipid classes LPE, ST, LPS, LPC, LPC(O-), LPA, LPS, Cer, CE, SM, PA, TG, PE, PI(O-), PGP-Me, PC(O-), PC, PS, FFA
Marković et al. (2024) [99]	LC-HRMS	SCH 30 HC 31	50 48.4	Between 24 and 74 years	untargeted analysis, 192 features, lipid classes PC, PC(O-), LPC, PS, PI, LPI, LPS(O-), PA, SM, Cer, DG, TG, MG, FFA, CE	differential lipids male 49, female 60 upregulated in both genders Cer(34:1), Cer(34:2), LPC(16:0), TG(48:2)	
Shi et al. (2024) [100]	UPLC-MS/MS	SCH 96 HC 96	male	40.43 (9.60) 40.52 (9.46)	untargeted analysis, 1033 lipids, FFA, GL, GP, SP, ST, isopentols	34 differential lipids, 21 downregulated, 13 upregulated, 10 lipid species were associated with cognition	PIs ↑, all PE(O-) ↓, all PE(P-) ↓

Mean ages * were presented as average value(stdev) or median value(1st, 3rd quartile); LC—liquid chromatography; MS—mass spectrometry; HPLC—high-pressure LC; UHPLC—ultra-HPLC; MS/MS—MS coupled with MS (tandem MS); BD—bipolar disorder; HC—healthy controls; SCH—schizophrenia; MDD—major depression disorder; aa—acylacyl; ae—acylalkyl; acyl-CAR—acyl-carnitines; CAR—carnitine; CE—cholesterol ester; Cer—ceramide; CerP—ceramide-phosphates; DG—diacylglicerols; FFA—free fatty acids; GD2—ganglioside GD2; GL—glycerolipids; GluCer—glucosyl-Cer; GM2—ganglioside GM2; GP—glycerophospholipids; LacCer-lactosyl-Cer; LPA—lysophosphatidic acid; LPC—lysophosphatidyl-choline; LPC(O-)—ether-LPC; LPC(P-)—LPC-plasmalogen; LPE—lysophosphatidyl-ethanolamine; LPI—lysophosphatidyl-inositol; LPS(O-)—ether-LPS; MG—monoacylglycerols; MGDG—monogalactosyldiacylglycerols; LPS—lysophosphatidyl-serine; PA—phosphatidic acid; PA(O-)-ether-PA; PC—phosphatidyl-choline; PC(O-)—ether-PC; PC(P-)—PC plasmalogen; PE—phosphatidyl-ethanolamine; PE(O-)—ether-PE; PE(P-)—PE plasmalogen PE; PG—phosphatidyl-glycerol; PG(O-)—ether-PG; PI—phosphatidyl-inositol; PI(O-)—ether-PI; PIP—phospho-phosphatidyl-inositol; PIP2—bisphospho-phosphatidyl-inositol; PS—phosphatidyl-serine; SM—sphingomyelin; SP—sphingolipids; ST—sterol lipids; TG—triacylglicerols; WE—wax-esters; ↑—upregulated; ↓—downregulated: ↔—remain unchanged.

## References

[1] Morrissette D.A., Stahl S.M. (2011). Affective symptoms in schizophrenia. Drug Discov. Today Ther. Strateg..

[2] Fountoulakis K.N., Popovic D., Mosheva M., Siamouli M., Moutou K., Gonda X. (2017). Mood symptoms in stabilized patients with schizophrenia: A bipolar type with predominant psychotic features?. Psychiatr. Danub..

[3] Berrettini W.H. (2000). Are schizophrenic and bipolar disorders related? A review of family and molecular studies. Biol. Psychiatry.

[4] Sorella S., Lapomarda G., Messina I., Frederickson J.J., Siugzdaite R., Job R., Grecucci A. (2019). Testing the expanded continuum hypothesis of schizophrenia and bipolar disorder. Neural and psychological evidence for shared and distinct mechanisms. Neuroimage Clin..

[5] Moreno-Küstner B., Martín C., Pastor L. (2018). Prevalence of psychotic disorders and its association with methodological issues. A systematic review and meta-analyses. PLoS ONE.

[6] Fasseeh A., Németh B., Molnár A., Fricke F.U., Horváth M., Kóczián K., Götze Á., Kaló Z. (2018). A systematic review of the indirect costs of schizophrenia in Europe. Eur. J. Public. Health.

[7] Desai P.R., Lawson K.A., Barner J.C., Rascati K.L. (2013). Estimating the direct and indirect costs for community-dwelling patients with schizophrenia. J. Pharmac. Health Serv. Res..

[8] Fajutrao L., Locklear J., Priaulx J., Heyes A. (2009). A systematic review of the evidence of the burden of bipolar disorder in Europe. Clin. Pract. Epidemiol. Ment. Health.

[9] Cloutier M., Greene M., Guerin A., Touya M., Wu E. (2018). The economic burden of bipolar I disorder in the United States in 2015. J. Affect. Disord..

[10] Barbosa W.B., Costa J.O., de Lemos L.L.P., Gomes R.M., de Oliveira H.N., Ruas C.M., Acurcio F.A., Barbui C., Bennie M., Godman B. (2018). Costs in the treatment of schizophrenia in adults receiving atypical antipsychotics: An 11-year cohort in Brazil. Appl. Health Econ. Health Policy..

[11] Zhu B., Ascher-Svanum H., Faries D.E., Peng X., Salkever D., Slade E.P. (2008). Costs of treating patients with schizophrenia who have illness-related crisis events. BMC Psychiatry.

[12] Yang R., Zhao Y., Tan Z., Lai J., Chen J., Zhang X., Sun J., Chen L., Lu K., Cao L. (2023). Differentiation between bipolar disorder and major depressive disorder in adolescents: From clinical to biological biomarkers. Front. Hum. Neurosci..

[13] Oliva V., Fico G., De Prisco M., Gonda X., Rosa A.R., Vieta E. (2024). Bipolar disorders: An update on critical aspects. Lancet Reg. Health Eur..

[14] World Health Organization Mental Disorders. World Health Organization; 2024. https://www.who.int/news-room/fact-sheets/detail/mental-disorders.

[15] Goldberg J.F. (2019). Complex combination pharmacotherapy for bipolar disorder: Knowing when less is more or more is better. Focus.

[16] Pillinger T., Howes O.D., Correll C.U., Leucht S., Huhn M., Schneider-Thoma J., Gaughran F., Jauhar S., McGuire P.K., Taylor D.M. (2023). Antidepressant and antipsychotic side-effects and personalised prescribing: A systematic review and digital tool development. Lancet Psychiatry.

[17] Ganse-Dumrath A., Chohan A., Samuel S., Bretherton P., Haenschel C., Fett A.K. (2025). Systematic review and meta-analysis of early visual processing, social cognition, and functional outcomes in schizophrenia spectrum disorders. Schizophr. Res. Cogn..

[18] Peritogiannis V., Ninou A., Samakouri M. (2022). Mortality in schizophrenia-spectrum disorders: Recent advances in understanding and management. Healthcare.

[19] Gogtay N., Vyas N.S., Testa R., Wood S.J., Pantelis C. (2011). Age of onset of schizophrenia: Perspectives from structural neuroimaging studies. Schizophr. Bull..

[20] Reyes-Lizaola S., Luna-Zarate U., Tendilla-Beltrán H., Morales-Medina J.C., Flores G. (2024). Structural and biochemical alterations in dendritic spines as key mechanisms for severe mental illnesses. Prog. Neuropsychopharmacol. Biol. Psychiatry.

[21] Lin Z., Long F., Kang R., Klionsky D.J., Yang M., Tang D. (2024). The lipid basis of cell death and autophagy. Autophagy.

[22] Santos A.L., Preta G. (2018). Lipids in the cell: Organisation regulates function. Cell Mol. Life Sci..

[23] Liu H., Wang S., Wang J., Guo X., Song Y., Fu K., Gao Z., Liu D., He W., Yang L.L. (2025). Energy metabolism in health and diseases. Signal Transduct. Target. Ther..

[24] Andersen C.J. (2022). Lipid metabolism in inflammation and immune function. Nutrients.

[25] Qiu S., Cai Y., Yao H., Lin C., Xie Y., Tang S., Zhang A. (2023). Small molecule metabolites: Discovery of biomarkers and therapeutic targets. Signal Transduct. Target. Ther..

[26] Bhargava S., de la Puente-Secades S., Schurgers L., Jankowski J. (2022). Lipids and lipoproteins in cardiovascular diseases: A classification. Trends Endocrinol. Metab..

[27] Zhu Y., Wan F., Liu J., Jia Z., Song T. (2024). The critical role of lipid metabolism in health and diseases. Nutrients.

[28] Zorkina Y., Ushakova V., Ochneva A., Tsurina A., Abramova O., Savenkova V., Goncharova A., Alekseenko I., Morozova I., Riabinina D. (2024). Lipids in psychiatric disorders: Functional and potential diagnostic role as blood biomarkers. Metabolites.

[29] Osetrova M., Tkachev A., Mair W., Guijarro Larraz P., Efimova O., Kurochkin I., Stekolshchikova E., Anikanov N., Foo J.C., Cazenave-Gassiot A. (2024). Lipidome atlas of the adult human brain. Nat. Commun..

[30] Delacrétaz A., Vandenberghe F., Gholam-Rezaee M., Saigi Morgui N., Glatard A., Thonney J., Solida-Tozzi A., Kolly S., Gallo S.F., Baumann P. (2018). Early changes of blood lipid levels during psychotropic drug treatment as predictors of long-term lipid changes and of new onset dyslipidemia. J. Clin. Lipidol..

[31] Network and Pathway Analysis Subgroup of Psychiatric Genomics Consortium (2015). Psychiatric genome-wide association study analyses implicate neuronal, immune and histone pathways. Nat. Neurosci..

[32] Dickens A.M., Sen P., Kempton M.J., Barrantes-Vidal N., Iyegbe C., Nordentoft M., Pollak T., Riecher-Rössler A., Ruhrmann S., Sachs G. (2021). Dysregulated lipid metabolism precedes onset of psychosis. Biol. Psychiatry.

[33] Ventriglio A., Bellomo A., Donato F., Iris B., Giovanna V., Dario D.S., Edwige C., Di Gioia I., Pettorruso M., Perna G. (2021). Oxidative stress in the early stage of psychosis. Curr. Top. Med. Chem..

[34] Vauzour D., Martinsen A., Layé S. (2015). Neuroinflammatory processes in cognitive disorders: Is there a role for flavonoids and n-3 polyunsaturated fatty acids in counteracting their detrimental effects?. Neurochem. Int..

[35] Borsini A., Nicolaou A., Camacho-Muñoz D., Kendall A.C., Di Benedetto M.G., Giacobbe J., Su K.P., Pariante C.M. (2021). Omega-3 polyunsaturated fatty acids protect against inflammation through production of LOX and CYP450 lipid mediators: Relevance for major depression and for human hippocampal neurogenesis. Mol. Psychiatry.

[36] Cobley J.N., Fiorello M.L., Bailey D.M. (2018). 13 reasons why the brain is susceptible to oxidative stress. Redox Biol..

[37] Salim S. (2017). Oxidative stress and the central nervous system. J. Pharmacol. Exp. Ther..

[38] Wei F., Lamichhane S., Orešič M., Hyötyläinen T. (2019). Lipidomes in health and disease: Analytical strategies and considerations. TrAC Trends Anal. Chem..

[39] Huang Y., Sulek K., Stinson S.E., Holm L.A., Kim M., Trost K., Hooshmand K., Lund M.A.V., Fonvig C.E., Juel H.B. (2025). Lipid profiling identifies modifiable signatures of cardiometabolic risk in children and adolescents with obesity. Nat. Med..

[40] Bellot P.E.N.R., Braga E.S., Omage F.B., da Silva Nunes F.L., Lima S.C.V.C., Lyra C.O., Marchioni D.M.L., Pedrosa L.F.C., Barbosa F., Tasic L. (2023). Plasma lipid metabolites as potential biomarkers for identifying individuals at risk of obesity-induced metabolic complications. Sci. Rep..

[41] Costa A.C., Riça L.B., van de Bilt M., Zandonadi F.S., Gattaz W.F., Talib L.L., Sussulini A. (2023). Application of lipidomics in psychiatry: Plasma-based potential biomarkers in schizophrenia and bipolar disorder. Metabolites.

[42] Sethi S., Hayashi M.A., Sussulini A., Tasic L., Brietzke E. (2017). Analytical approaches for lipidomics and its potential applications in neuropsychiatric disorders. World J. Biol. Psychiatry.

[43] Yang K., Han X. (2016). Lipidomics: Techniques, applications, and outcomes related to biomedical sciences. Trends Biochem. Sci..

[44] Kim D.H., Kim Y.S., Son N.I., Kang C.K., Kim A.R. (2017). Recent omics technologies and their emerging applications for personalised medicine. IET Syst. Biol..

[45] Medina J., Goss N., Correia G.D.S., Borreggine R., Teav T., Kutalik Z., Vidal P.M., Gallart-Ayala H., Ivanisevic J. (2025). Clinical lipidomics reveals high individuality and sex specificity of circulatory lipid signatures: A prospective healthy population study. J. Lipid Res..

[46] Züllig T., Trötzmüller M., Köfeler H.C. (2020). Lipidomics from sample preparation to data analysis: A primer. Anal. Bioanal. Chem..

[47] Quehenberger O., Armando A.M., Brown A.H., Milne S.B., Myers D.S., Merrill A.H., Bandyopadhyay S., Jones K.N., Kelly S., Shaner R.L. (2010). Lipidomics reveals a remarkable diversity of lipids in human plasma. J. Lipid Res..

[48] Wang D., Cheng S.L., Fei Q., Gu H., Raftery D., Cao B., Sun X., Yan J., Zhang C., Wang J. (2019). Metabolic profiling identifies phospholipids as potential serum biomarkers for schizophrenia. Psychiatry Res..

[49] Yan L., Zhou J., Wang D., Si D., Liu Y., Zhong L., Yin Y. (2018). Unbiased lipidomic profiling reveals metabolomic changes during the onset and antipsychotics treatment of schizophrenia disease. Metabolomics.

[50] Tkachev A., Stekolshchikova E., Vanyushkina A., Zhang H., Morozova A., Zozulya S., Kurochkin I., Anikanov N., Egorova A., Yushina E. (2023). Lipid alteration signature in the blood plasma of Individuals with schizophrenia, depression, and bipolar disorder. JAMA Psychiatry.

[51] Guo L., Zhang T., Li R., Cui Z.Q., Du J., Yang J.B., Xue F., Chen Y.H., Tan Q.R., Peng Z.W. (2022). Alterations in the plasma lipidome of adult women with bipolar disorder: A mass spectrometry-based lipidomics research. Front. Psychiatry.

[52] Hone E., Lim F., Martins I.J., Martins R.N., Brennan C.S., Binosha Fernando W.M.A.D., Brennan M.A., Fuller S.J. (2019). Neurodegeneration and Alzheimer’s Disease: The Role of Diabetes, Genetics, Hormones, and Lifestyle.

[53] Hsu M.C., Ouyang W.C. (2021). A systematic review of effectiveness of omega-3 fatty acid supplementation on symptoms, social functions, and neurobiological variables in schizophrenia. Biol. Res. Nurs..

[54] Goh K.K., Chen C.Y., Chen C.H., Lu M.L. (2021). Effects of omega-3 polyunsaturated fatty acids supplements on psychopathology and metabolic parameters in schizophrenia: A meta-analysis of randomized controlled trials. J. Psychopharmacol..

[55] Lv S., Luo C. (2025). Ferroptosis in schizophrenia: Mechanisms and therapeutic potentials. Mol. Med. Rep..

[56] Murray A.J., Rogers J.C., Katshu M.Z.U.H., Liddle P.F., Upthegrove R. (2021). Oxidative stress and the pathophysiology and symptom profile of schizophrenia spectrum disorders. Front. Psychiatry.

[57] Seabra G., Falvella A.C.B., Guest P.C., Martins-de-Souza D., de Almeida V. (2018). Proteomics and lipidomics in the elucidation of endocannabinoid signaling in healthy and schizophrenia brains. Proteomics.

[58] Messinis A., Panteli E., Paraskevopoulou A., Zymarikopoulou A.K., Filiou M.D. (2024). Altered lipidomics biosignatures in schizophrenia: A systematic review. Schizophr. Res..

[59] Wu S., Panganiban K.J., Lee J., Li D., Smith E.C.C., Maksyutynska K., Humber B., Ahmed T., Agarwal S.M., Ward K. (2024). Peripheral lipid signatures, metabolic dysfunction, and pathophysiology in schizophrenia spectrum disorders. Metabolites.

[60] Hiller J.K., Jangmo A., Tesli M.S., Jaholkowski P.P., Hoseth E.Z., Steen N.E., Haram M. (2023). Lipid biomarker research in bipolar disorder: A scoping review of trends, challenges, and future directions. Biol. Psychiatry Glob. Open Sci..

[61] Schwarz E., Prabakaran S., Whitfield P., Major H., Leweke F.M., Koethe D., McKenna P., Bahn S. (2008). High throughput lipidomic profiling of schizophrenia and bipolar disorder brain tissue reveals alterations of free fatty acids, phosphatidylcholines, and ceramides. J. Proteome Res..

[62] McNamara R.K., Rider T., Jandacek R., Tso P. (2014). Abnormal fatty acid pattern in the superior temporal gyrus distinguishes bipolar disorder from major depression and schizophrenia and resembles multiple sclerosis. Psychiatry Res..

[63] Ribeiro H.C., Klassen A., Pedrini M., Carvalho M.S., Rizzo L.B., Noto M.N., Zeni-Graiff M., Sethi S., Fonseca F.A.H., Tasic L. (2017). A preliminary study of bipolar disorder type I by mass spectrometry-based serum lipidomics. Psychiatry Res..

[64] Brunkhorst-Kanaan N., Klatt-Schreiner K., Hackel J., Schröter K., Trautmann S., Hahnefeld L., Wicker S., Reif A., Thomas D., Geisslinger G. (2019). Targeted lipidomics reveal derangement of ceramides in major depression and bipolar disorder. Metabolism..

[65] Zhang T., Guo L., Li R., Wang F., Yang W.M., Yang J.B., Cui Z.Q., Zhou C.H., Chen Y.H., Yu H. (2022). Alterations of plasma lipids in adult women with major depressive disorder and bipolar depression. Front. Psychiatry.

[66] Jadranin M., Avramović N., Miladinović Z., Gavrilović A., Tasic L., Tešević V., Mandić B. (2023). Untargeted lipidomics study of bipolar disorder patients in Serbia. Int. J. Mol. Sci..

[67] Clegg D. (2012). Minireview: The year in review of estrogen regulation of metabolism. Mol. Endocrin..

[68] Gilroy D.W., Bishop-Bailey D. (2019). Lipid mediators in immune regulation and resolution. Br. J. Pharmacol..

[69] Alonso N., Zelzer S., Eibinger G., Herrmann M. (2023). Vitamin D metabolites: Analytical challenges and clinical relevance. Calcif. Tissue Int..

[70] İmre O., Karaağaç M., Caglayan C. (2023). Does decreased vitamin D level trigger bipolar manic attacks?. Behav. Sci..

[71] Meinhard N., Kessing L.V., Vinberg M. (2014). The role of estrogen in bipolar disorder, a review. Nord. J. Psychiatry.

[72] Gurvich A., Begemann M., Dahm L., Sargin D., Miskowiak K., Ehrenreich H. (2014). A role for prostaglandins in rapid cycling suggested by episode-specific gene expression shifts in peripheral blood mononuclear cells: A preliminary report. Bipolar. Disord..

[73] Hahn C.G., Friedman E. (1999). Abnormalities in protein kinase C signaling and the pathophysiology of bipolar disorder. Bipolar Disord..

[74] Knowles E.E., Meikle P.J., Huynh K., Göring H.H., Olvera R.L., Mathias S.R., Duggirala R., Almasy L., Blangero J., Curran J.E. (2017). Serum phosphatidylinositol as a biomarker for bipolar disorder liability. Bipolar Disord..

[75] Ju S., Greenberg M.L. (2003). Valproate disrupts regulation of inositol responsive genes and alters regulation of phospholipid biosynthesis. Mol. Microbiol..

[76] Chung K.H., Tsai S.Y., Lee H.C. (2007). Mood symptoms and serum lipids in acute phase of bipolar disorder in Taiwan. Psychiatry Clin. Neurosci..

[77] Wysokiński A., Strzelecki D., Kłoszewska I. (2015). Levels of triglycerides, cholesterol, LDL, HDL and glucose in patients with schizophrenia, unipolar depression and bipolar disorder. Diabetes Metab. Syndr..

[78] Kornhuber J., Tripal P., Reichel M., Mühle C., Rhein C., Muehlbacher M., Groemer T.W., Gulbins E. (2010). Functional inhibitors of acid sphingomyelinase (FIASMAs): A novel pharmacological group of drugs with broad clinical applications. Cell Physiol. Biochem..

[79] Gulbins E., Palmada M., Reichel M., Lüth A., Böhmer C., Amato D., Müller C.P., Tischbirek C.H., Groemer T.W., Tabatabai G. (2013). Acid sphingomyelinase-ceramide system mediates effects of antidepressant drugs. Nat. Med..

[80] Grassmé H., Jernigan P.L., Hoehn R.S., Wilker B., Soddemann M., Edwards M.J., Müller C.P., Kornhuber J., Gulbins E. (2015). Inhibition of acid sphingomyelinase by antidepressants counteracts stress-induced activation of P38-kinase in major depression. Neurosignals.

[81] Rao R.P., Yuan C., Allegood J.C., Rawat S.S., Edwards M.B., Wang X., Merrill A.H., Acharya U., Acharya J.K. (2007). Ceramide transfer protein function is essential for normal oxidative stress response and lifespan. Proc. Natl. Acad. Sci. USA.

[82] Mantovani A., Altomari A., Lunardi G., Bonapace S., Lippi G., Bonnet F., Targher G. (2020). Association between specific plasma ceramides and high-sensitivity C-reactive protein levels in postmenopausal women with type 2 diabetes. Diabetes Metab..

[83] Juchnicka I., Kuźmicki M., Zabielski P., Krętowski A., Błachnio-Zabielska A., Szamatowicz J. (2022). Serum C18:1-Cer as a potential biomarker for early detection of gestational diabetes. J. Clin. Med..

[84] Yu B., Hu M., Jiang W., Ma Y., Ye J., Wu Q., Guo W., Sun Y., Zhou M., Xu Y. (2023). Ceramide d18:1/24:1 as a potential biomarker to differentiate obesity subtypes with unfavorable health outcomes. Lipids Health Dis..

[85] Shu H., Peng Y., Hang W., Li N., Zhou N., Wang D.W. (2022). Emerging roles of ceramide in cardiovascular diseases. Aging Dis..

[86] Harris E. (2024). First-line antidepressants tied to minor differences in weight gain. JAMA.

[87] Tomasik J., Harrison S.J., Rustogi N., Olmert T., Barton-Owen G., Han S.Y.S., Cooper J.D., Eljasz P., Farrag L.P., Friend L.V. (2024). Metabolomic biomarker signatures for bipolar and unipolar depression. JAMA Psychiatry.

[88] Orešič M., Seppänen-Laakso T., Sun D., Tang J., Therman S., Viehman R., Mustonen U., van Erp T.G., Hyötyläinen T., Thompson P. (2012). Phospholipids and insulin resistance in psychosis: A lipidomics study of twin pairs discordant for schizophrenia. Genome Med..

[89] Orešič M., Tang J., Seppänen-Laakso T., Mattila I., Saarni S.E., Saarni S.I., Lönnqvist J., Sysi-Aho M., Hyötyläinen T., Perälä J. (2011). Metabolome in schizophrenia and other psychotic disorders: A general population-based study. Genome Med..

[90] Kaddurah-Daouk R., McEvoy J., Baillie R.A., Lee D., Yao J.K., Doraiswamy P.M., Krishnan K.R. (2007). Metabolomic mapping of atypical antipsychotic effects in schizophrenia. Mol. Psychiatry.

[91] Kaddurah-Daouk R., McEvoy J., Baillie R., Zhu H., Yao J.K., Nimgaonkar V.L., Buckley P.F., Keshavan M.S., Georgiades A., Nasrallah H.A. (2012). Impaired plasmalogens in patients with schizophrenia. Psychiatry Res..

[92] McEvoy J., Baillie R.A., Zhu H., Buckley P., Keshavan M.S., Nasrallah H.A., Dougherty G.G., Yao J.K., Kaddurah-Daouk R. (2013). Lipidomics reveals early metabolic changes in subjects with schizophrenia: Effects of atypical antipsychotics. PLoS ONE.

[93] Wood P.L., Unfried G., Whitehead W., Phillipps A., Wood J.A. (2015). Dysfunctional plasmalogen dynamics in the plasma and platelets of patients with schizophrenia. Schizophr. Res..

[94] Hylén U., McGlinchey A., Orešič M., Bejerot S., Humble M.B., Särndahl E., Hyötyläinen T., Eklund D. (2021). Potential transdiagnostic lipid mediators of inflammatory activity in individuals with serious mental illness. Front. Psychiatry.

[95] Cao B., Wang D., Pan Z., Brietzke E., McIntyre R.S., Musial N., Mansur R.B., Subramanieapillai M., Zeng J., Huang N. (2019). Characterizing acyl-carnitine biosignatures for schizophrenia: A longitudinal pre- and post-treatment study. Transl. Psychiatry.

[96] Leppik L., Parksepp M., Janno S., Koido K., Haring L., Vasar E., Zilmer M. (2020). Profiling of lipidomics before and after antipsychotic treatment in first-episode psychosis. Eur. Arch. Psychiatry Clin. Neurosci..

[97] Wang D., Sun X., Maziade M., Mao W., Zhang C., Wang J., Cao B. (2021). Characterising phospholipids and free fatty acids in patients with schizophrenia: A case-control study. World J. Biol. Psychiatry.

[98] Wang F., Guo L., Zhang T., Cui Z., Wang J., Zhang C., Xue F., Zhou C., Li B., Tan Q. (2022). Alterations in plasma lipidomic profiles in adult patients with schizophrenia and major depressive disorder. Medical.

[99] Marković S., Jadranin M., Miladinović Z., Gavrilović A., Avramović N., Takić M., Tasic L., Tešević V., Mandić B. (2024). LC-HRMS lipidomic fingerprints in Serbian cohort of schizophrenia patients. Int. J. Mol. Sci..

[100] Shi M., Du X., Jia Y., Zhang Y., Jia Q., Zhang X., Zhu Z. (2024). The identification of novel schizophrenia-related metabolites using untargeted lipidomics. Cereb. Cortex..

[101] Longo N., Frigeni M., Pasquali M. (2016). Carnitine transport and fatty acid oxidation. Biochim. Biophys. Acta..

[102] Ferreira G.C., McKenna M.C. (2017). L-carnitine and acetyl-L-carnitine roles and neuroprotection in developing brain. Neurochem. Res..

[103] Steiber A., Kerner J., Hoppel C.L. (2004). Carnitine: A nutritional, biosynthetic, and functional perspective. Mol. Asp. Med..

[104] Jones L.L., McDonald D.A., Borum P.R. (2010). Acylcarnitines: Role in brain. Prog. Lipid Res..

[105] Zhao L., Liu H., Wang W., Wang Y., Xiu M., Li S. (2023). Carnitine metabolites and cognitive improvement in patients with schizophrenia treated with olanzapine: A prospective longitudinal study. Front. Pharmacol..

[106] Schooneman M.G., Vaz F.M., Houten S.M., Soeters M.R. (2013). Acylcarnitines: Reflecting or inflicting insulin resistance?. Diabetes..

[107] Lheureux P.E., Penaloza A., Zahir S., Gris M. (2005). Science review: Carnitine in the treatment of valproic acid-induced toxicity-what is the evidence?. Crit. Care..

[108] Bene J., Hadzsiev K., Melegh B. (2018). Role of carnitine and its derivatives in the development and management of type 2 diabetes. Nutr. Diabetes.

[109] Wang W., Pan D., Liu Q., Chen X., Wang S. (2024). L-carnitine in the treatment of psychiatric and neurological manifestations: A systematic review. Nutrients.

[110] Berger G.E., Wood S.J., Pantelis C., Velakoulis D., Wellard R.M., McGorry P.D. (2002). Implications of lipid biology for the pathogenesis of schizophrenia. Aust. N. Z. J. Psychiatry.

[111] Prabutzki P., Schiller J., Engel K.M. (2024). Phospholipid-derived lysophospholipids in (patho)physiology. Atherosclerosis.

[112] Stadler J.T., Bärnthaler T., Borenich A., Emrich I.E., Habisch H., Rani A., Holzer M., Madl T., Heine G.H., Marsche G. (2024). Low LCAT activity is linked to acute decompensated heart failure and mortality in patients with CKD. J. Lipid Res..

[113] Knuplez E., Marsche G. (2020). An updated review of pro- and anti-inflammatory properties of plasma lysophosphatidylcholines in the vascular system. Int. J. Mol. Sci..

[114] Hossain M.S., Mawatari S., Fujino T. (2020). Biological functions of plasmalogens. Adv. Exp. Med. Biol..

[115] Udagawa J., Hino K. (2022). Plasmalogen in the brain: Effects on cognitive functions and behaviors attributable to its properties. Brain Res. Bull..

[116] Bozelli J.C., Azher S., Epand R.M. (2021). Plasmalogens and chronic inflammatory diseases. Front. Physiol..

[117] Ferreri C., Ferocino A., Batani G., Chatgilialoglu C., Randi V., Riontino M.V., Vetica F., Sansone A. (2023). Plasmalogens: Free radical reactivity and identification of trans isomers relevant to biological membranes. Biomolecules.

[118] Kriisa K., Leppik L., Balõtšev R., Ottas A., Soomets U., Koido K., Volke V., Innos J., Haring L., Vasar E. (2017). Profiling of acylcarnitines in first episode psychosis before and after antipsychotic treatment. J. Proteome Res..

[119] Lauer A.A., Griebsch L.V., Pilz S.M., Janitschke D., Theiss E.L., Reichrath J., Herr C., Beisswenger C., Bals R., Valencak T.G. (2021). Impact of vitamin D_3_ deficiency on phosphatidylcholine-/ethanolamine, plasmalogen-, lyso-phosphatidylcholine-/ethanolamine, carnitine- and triacyl glyceride-homeostasis in neuroblastoma cells and murine brain. Biomolecules.

[120] Sun G.Y., Xu J., Jensen M.D., Yu S., Wood W.G., González F.A., Simonyi A., Sun A.Y., Weisman G.A. (2005). Phospholipase A2 in astrocytes: Responses to oxidative stress, inflammation, and G protein-coupled receptor agonists. Mol. Neurobiol..

[121] Walther A., Cannistraci C.V., Simons K., Durán C., Gerl M.J., Wehrli S., Kirschbaum C. (2018). Lipidomics in major depressive disorder. Front. Psychiatry.

[122] Decker S.T., Funai K. (2024). Mitochondrial membrane lipids in the regulation of bioenergetic flux. Cell Metab..

[123] Cole L.K., Vance J.E., Vance D.E. (2012). Phosphatidylcholine biosynthesis and lipoprotein metabolism. Biochim. Biophys. Acta..

[124] Simić K., Miladinović Z., Todorović N., Trifunović S., Avramović N., Gavrilović A., Jovanović S., Gođevac D., Vujisić L., Tešević V. (2023). Metabolomic profiling of bipolar disorder by ^1^H-NMR in Serbian patients. Metabolites.

[125] Simić K., Todorović N., Trifunović S., Miladinović Z., Gavrilović A., Jovanović S., Avramović N., Gođevac D., Vujisić L., Tešević V. (2022). NMR metabolomics in serum fingerprinting of schizophrenia patients in a Serbian cohort. Metabolites.

[126] Tkachev A., Stekolshchikova E., Anikanov N., Zozulya S., Barkhatova A., Klyushnik T., Petrova D. (2021). Shorter chain triglycerides are negatively associated with symptom improvement in schizophrenia. Biomolecules.

[127] Li J., Wang F., Xue R., Si S., Tang F., Xue F. (2022). Effects of antipsychotics on triglyceride trajectories and its implications in CVD: A longitudinal cohort study. EBioMedicine.

[128] Skrede S., Steen V.M., Fernø J. (2013). Antipsychotic-induced increase in lipid biosynthesis: Activation through inhibition?. J. Lipid Res..

[129] Hanamatsu H., Ohnishi S., Sakai S., Yuyama K., Mitsutake S., Takeda H., Hashino S., Igarashi Y. (2014). Altered levels of serum sphingomyelin and ceramide containing distinct acyl chains in young obese adults. Nutr. Diabetes..

[130] Turpin-Nolan S.M., Brüning J.C. (2020). The role of ceramides in metabolic disorders: When size and localization matters. Nat. Rev. Endocrinol..

[131] Subbaiah P.V., Jiang X.C., Belikova N.A., Aizezi B., Huang Z.H., Reardon C.A. (2012). Regulation of plasma cholesterol esterification by sphingomyelin: Effect of physiological variations of plasma sphingomyelin on lecithin-cholesterol acyltransferase activity. Biochim. Biophys. Acta..

[132] Yang F., Chen G. (2022). The nutritional functions of dietary sphingomyelin and its applications in food. Front. Nutr..

[133] Gault C.R., Obeid L.M., Hannun Y.A. (2010). An overview of sphingolipid metabolism: From synthesis to breakdown. Adv. Exp. Med. Biol..

[134] Rodriguez-Cuenca S., Pellegrinelli V., Campbell M., Oresic M., Vidal-Puig A. (2017). Sphingolipids and glycerophospholipids-The “ying and yang” of lipotoxicity in metabolic diseases. Prog. Lipid Res..

[135] Tao S., Zhang Y., Wang Q., Qiao C., Deng W., Liang S., Wei J., Wei W., Yu H., Li X. (2022). Identifying transdiagnostic biological subtypes across schizophrenia, bipolar disorder, and major depressive disorder based on lipidomics profiles. Front. Cell Dev. Biol..

[136] Yu L., Long Q., Zhang Y., Liu Y., Guo Z., Cao X., Qin F., Xu Y., Qian Q., Gao B. (2024). Bidirectional Mendelian randomization analysis of plasma lipidome and psychiatric disorders. J. Affect. Disord..

[137] Bernal-Vega S., García-Juárez M., Camacho-Morales A. (2023). Contribution of ceramides metabolism in psychiatric disorders. J. Neurochem..

[138] Sfera A., Imran H., Sfera D.O., Anton J.J., Kozlakidis Z., Hazan S. (2024). Novel Insights into psychosis and antipsychotic interventions: From managing symptoms to improving outcomes. Int. J. Mol. Sci..

[139] Ottensmann L., Tabassum R., Ruotsalainen S.E., Gerl M.J., Klose C., Widén E., FinnGen, Simons K., Ripatti S., Pirinen M. (2023). Genome-wide association analysis of plasma lipidome identifies 495 genetic associations. Nat. Commun..

